# 3D bioprinting for the construction of drug testing models-development strategies and regulatory concerns

**DOI:** 10.3389/fbioe.2025.1457872

**Published:** 2025-02-14

**Authors:** Divya Mallya, Mrunmayi Ashish Gadre, S. Varadharajan, Kirthanashri S. Vasanthan

**Affiliations:** ^1^ Manipal Centre for Biotherapeutics Research, Manipal Academy of Higher Education, Manipal, Karnataka, India; ^2^ Manipal Institute of Technology, Manipal Academy of Higher Education, Manipal, Karnataka, India

**Keywords:** additive manufacturing, bioengineering, 3D bioprinting, drug testing 3D models, drug screening and discovery

## Abstract

A drug to be successfully launched in the market requires a significant amount of capital, resources and time, where the unsuccessful results in the last stages lead to catastrophic failure for discovering drugs. This is the very reason which calls for the invention of innovative models that can closely mimic the human *in vivo* model for producing reliable results. Throughout the innovation line, there has been improvement in the rationale *in silico* designing but yet there is requirement for *in vitro-in vivo* correlations. During the evolving of the drug testing models, the 3D models produced by different methods have been proven to produce better results than the traditional 2D models. However, the *in vitro* fabrications of live tissues are still bottleneck in realizing their complete potential. There is an urgent need for the development of single, standard and simplified *in vitro* 3D tissue models that can be reliable for investigating the biological and pathological aspects of drug discovery, which is yet to be achieved. The existing pre-clinical models have considerable drawbacks despite being the gold standard in pre-clinical research. The major drawback being the interspecies differences and low reliability on the generated results. This gap could be overcome by the fabrication of bioengineered human disease models for drug screening. The advancement in the fabrication of 3D models will provide a valuable tool in screening drugs at different stages as they are one step closer to bio-mimic human tissues. In this review, we have discussed on the evolution of preclinical studies, and different models, including mini tissues, spheroids, organoids, bioengineered three dimensional models and organs on chips. Furthermore, we provide details of different disease models fabricated across various organs and their applications. In addition to this, the review also focuses on the limitations and the current prospects of the role of three dimensionally bioprinted models in drug screening and development.

## 1 Introduction

Scientific and medical advances have yielded innovative pharmaceuticals and therapies for various diseases. The scientific breakthrough of 3D bioprinting has advanced our understanding of disease mechanisms and drug effectiveness through its applications in tissue engineering and the creation of artificial organs. The field of pharmaceutical research can significantly improve by using 3D bioprinted models for testing drugs and disease modeling. This approach brings ethical advantages and improves accuracy. During a drug discovery, two approaches are involved which has the classical pharmacology that is the knowledge, practicing based on the existing historical basis of drug discovery and reverse pharmacology that is designated for employing the target-based drug discovery. The emerging concept of 3D model has been moving towards such therapeutic area which has proved to have promising *in vitro* results (Loewa et al., 2023). Historically, simple 2D cell cultures and animal models were used for drug screening and toxicity evaluation. Limitations in predicting human drug responses within these models have driven the development of more precise alternatives. Hence, 3D bioprinted tissues and organs that provide a more representative platform are being developed and employed for these studies. 3D bioprinting is an additive manufacturing technology in which bioartificial organs are created by layer-by-layer deposition of bioink composed of cells and biomaterials guided by a computer-aided design (CAD) model (Song et al., 2021). The various advantages of 3D bioprinting include control over cell distribution, high resolution of cell deposition, scalability, and cost-effectiveness (Mandrycky et al., 2016). In addition to cells, other tissue constituents like the extracellular matrix (ECM), growth factors, and other biomolecules can be included in the bioink and, ultimately, in the developed construct (Tan et al., 2021). Integrating 3D bioprinting, drug testing, and disease modeling can help replicate complex organ-drug interactions while adding other properties, such as vascularization, in structured and reproducible bioartificial organs. The 3D bioprinted drug testing models can help in bridging the gap between the interspecies differences along with poor prediction due to lack of complete human physiology and the high-end results with clinical mimicry (Lage et al., 2018). By incorporating 3D bioprinted entities within multi-well plates, it becomes feasible to conduct medium- and even high-throughput drug screening (Hagenbuchner et al., 2021). As scientific progress advances, 3D bioprinting in drug testing and disease modeling can reshape pharmaceutical research, contributing to the development of more efficacious, ethical, and personalized therapeutics.

## 2 Scenario on drug discovery and screening

### 2.1 The evolution of *in vitro* methods of drug screening

The history of mankind has been linked with ultimate use and exploration of various natural and bioactive substances, and the first ever medicinal drugs were plant-based including the herbs, vines and fungi (Dias et al., 2012). The use of natural products dates to the civilization based in areas like China, Egypt, Greek and Mesopotamia (Jones, 2011). Eventually by the 2000s, there was discontinuation of the use of traditional natural products screening due to the re-discovery of similar products and technical difficulties associated with the isolation of extracts (Lage et al., 2018). With the advancement in technology and instrumentation, there is rapid rate for identification of novel bioactive structures which has raised exigency for screening of such compounds (Bade et al., 2010). The drug screening includes a variety of analysis which assesses the molecular and biological extracts present in the drug and these assays are being carried out at different levels like the molecular, cellular, animal and clinical trials. There are two kinds of complementary approaches for discovering drugs which are applied - classic pharmacology which is based on the history of drug discovery that also involves the reverse pharmacology along with the target-based drug discovery, and the phenotypic drug discovery which involves the analysis of the molecular extracts assessed against a particular disease or disorder in a quantitative measure (Lee et al., 2012; Horman, 2016). In an experiment dating back to the 16th century, Sir William Harvey observed that a piece of myocardium covered in his saliva remained contractile for an extended period while placed on his palm. This experiment marked an important milestone that led to the development of cultured cell lines (Harvey, 2001). In 1910, Rous and team were successful in cell transformation by inducing tumour in cell line using chicken tumour cell extract (Rous sarcoma virus) which marked the landmark for the discovery of *in vitro* immortalization leading to the possibility of cell model production (Rous, 1910). Further transformation and improvement in research techniques lead to the use of higher organism models. Different models that have been successfully established for their potential in drug screening are listed in Table 1 along with the industries involved in production of these models. Model organisms involved in the drug discovery are the fruit fly, zebrafish, mouse and monkey which includes the whole animal level closed to the human genomics (Wheeler et al., 2012). Big animals that are used for several preclinical studies include mice, rats, dogs and monkeys. Approximately a century ago, the use of small rodents was considered as model organisms for primary research in the field of biomedicine. With the emergence of pharmaceutical industry, the use of rodents played an important role in the initial drug testing procedure (Frommlet and Heinze, 2021; Hartung, 2024). Over the years, results that have been obtained from the pre-clinical trials have a void due to interspecies and this gap can be filled by the *in vitro* models as they have capability to closely mimic human microenvironment as these models contain human cell lines (Satpathy et al., 2018; Foster and Putos, 2014; Aban and George, 2015). According to Newman and Cragg, the failure of clinical phases may be due to the lack of validations in preclinical stages and the insufficient availability of *in vitro* and *in vivo* disease models (Newman and Cragg, 2012). With the growing knowledge of the upcoming various *in vitro* and *in vivo* disease models, the rate of success in the development of wide range of drugs with accomplishing results will be higher, thus promising models would get established in the near future.

**TABLE 1:** Different tissue models applied for drug testing in 21st century.

Models	Image	Tissues/organs developed	Industries involved in production	References
2D cell culture	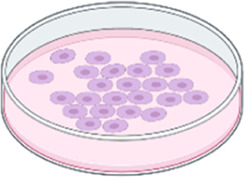	Epithelial Tissues, Immortalized Cell Cultures, Cancer Research, Stem Cell Maintenance and Differentiation	LifeGel, Biocellion/CMMC, Cocuus, Seawith Inc., Marinas Bio, BIOMILQ Inc., Brinter Inc	Kim, J et al. (2018), Duan et al. (2018), Cardoso et al. (2023)
Spheroid	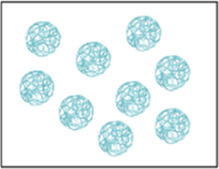	Cancer Models, Liver Tissues, Neural Tissue, Kidney Tissue, Pancreatic Islets and Cardiac Tissue.	Cyprotex, InSphero, n3DBiosciences, Sanbio	Loessner et al. (2010), Kim J et al. (2018), Mehta et al. (2024)
Organ-on-a-chip	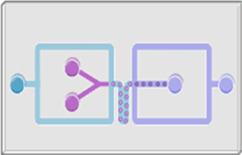	Lung, liver, heart, kidney and brain	AnaBios, IIVS, Visikol	Mittler et al. (2017), Wang H et al. (2018)
Reconstructed human tissue	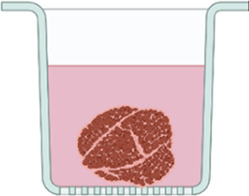	Skin tissues, corneal tissues, vascular tisues, bone tissues, cartiledge tissues and liver tissues	Cyprotex, EPISKIN, Epithelix, ImmuONE, J-TEC, MatTek, Phenion, ZenBio	Bunton (2011), Njoroge et al. (2021), Zhang X et al. (2022)
Organoid	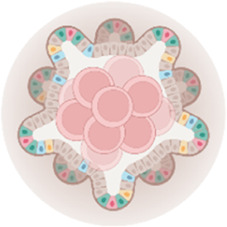	Intestines, brain, kidney, liver, pancreas and gastric organoids	Bio-Techne, Cellesce, HUB Organoids, InnoSer, Organoid Therapeutics, UPM Biomedicals	Vlachogiannis et al. (2018), Scognamiglio et al. (2019), Tebon et al. (2023)
3D Bioprinted model	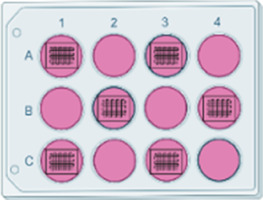	Skin, cartilage, bone, vascularized tissues, heart, liver and kidney	Aspect Biosystems, Cellbricks, CELLINK, Organovo, Cyfuse biomedical, EnvisionTEC, 3D bio Holdings Inc., Biobots, Stratasys, Precise bio, 3D systems Corp., 3D Biopritning solutions, 3D Bioteck, Adanced BioMatrix, Advanced Solution, Lifesciences, MicroFab, Technologies Inc., InSphero Inc., SHINING 3D TECH, 3D Systems, Avita Medical, Bespoke Innovations	Breathwaite et al. (2020), Tebon et al. (2023), Esser et al. (2023), Mazrouei et al. (2020), Muwaffak et al. (2017), Thompson (2023)

### 2.2 Current status in drug discovery

**FIGURE 1:**
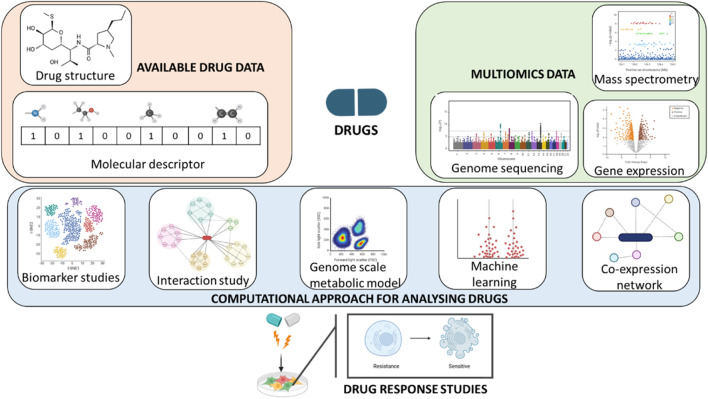
Computational and multi-omics analyses for drug discovery and development.

The rates of clinical trial and the drug discovery till date have low success rates as the process is time consuming and expensive with lot of ethical concerns. At present, due to the lack of efficacy, more than half of drugs do not pass the first and the second phase of clinical trials and one third of the drugs do not pass the clinical trials due to safety concerns and low therapeutic index (Arrowsmith and Miller, 2013). With the increase in the demand for drug discovery, there is an increase in the need for newer technologies which improve the precision of drug discovery. Lately, there are two promising areas which are expected to provide higher success rates: the use of biomarkers along with targeted drugs to improve the precision medicine, and the availability of new models prior to clinical trials that can better replicate the microenvironment and the biology of *in vivo* targets. Early in the 1980s, the importance of the extracellular matrix (ECM) in cell behaviour was being researched, which is now utilized in developing 3D models. The ECM plays an important role in mimicking the tissues and is much closer to representing the *in vivo* environment when compared to traditional 2D monolayer models for drug screening (Pampaloni et al., 2007; Ravi et al., 2015). The change in the ECM compositions leads to various physical changes, leading to bidirectional changes in both the ECM and the cells (Hynes, 2014; Alcaraz et al., 2017; Zollinger and Smith, 2017). Hence, the incorporation of ECM in drug testing models will improve the signalling in the models and provide appropriate results related to the drug characteristics (Mastikhina et al., 2020; Phang et al., 2022; Kim et al., 2020; Janani et al., 2022; Miller et al., 2021; Tian et al., 2022). There is a paradigm shift in the biomedical research where the focus is approaching towards the centralised human disease models (Hynes, 2014). This shift is due to the increasing rates in failure of drug development process in the present day. The investments in the drug development have increased over the past decade - in 2021, $133 billon had been invested in the leading 12 biggest pharma companies for drug research and development, which sums up to 44% increase since 2016. By 2021, the drug debilitation rates in all time had raised to 95% (Alcaraz et al., 2017; Zollinger and Smith, 2017). There are different reasons why the most of the drugs fail in the clinical trials, and one of the main reasons is the translational gap (i.e., the jump of drugs into clinical trial directly from an animal model) (Arrowsmith and Miller, 2013; Pampaloni et al., 2007). There are times where the animal model results might fail to filter out the harmfulness and ineffectiveness of the tested drugs (Ravi et al., 2015). On the other hand, there also might be chances of elimination of potential drugs which fail to produce results in the preclinical trials. The reason for such failures in the preclinical trials is due to the poor resemblance of these animal models to human conditions, and thus having low prediction values. The integration of the two growing fields - computational biology and multi-omics analyses - has revolutionized the discovery and development of drugs over the period of time (Zhou and Zhong, 2017). This incorporation of technology has helped in easy understanding of disease mechanisms and to identify different therapeutic targets (Chávez-Hernández et al., 2023). The combination of various technology offers a holistic view of biological system (Singh N. et al., 2023). Computational biology provides different fields like genomics, epigenomics, proteomics, transcriptomics and metabolomics to blend together and enable the researchers to capture complex data required during discovery of drugs (Giraud, 2024). The integration of these fields in drug discovery and disease modelling is depicted in Figure 1.

## 3 3D models for drug discovery and development

### 3.1 Different approaches to build 3D *in vitro* models

There are multiple models generated for testing wide range of drugs with having different aspects to study. Table 2 is briefly comparing the different models that are being used for testing drugs.

**TABLE 2:** Comparison of different models for drug testing.

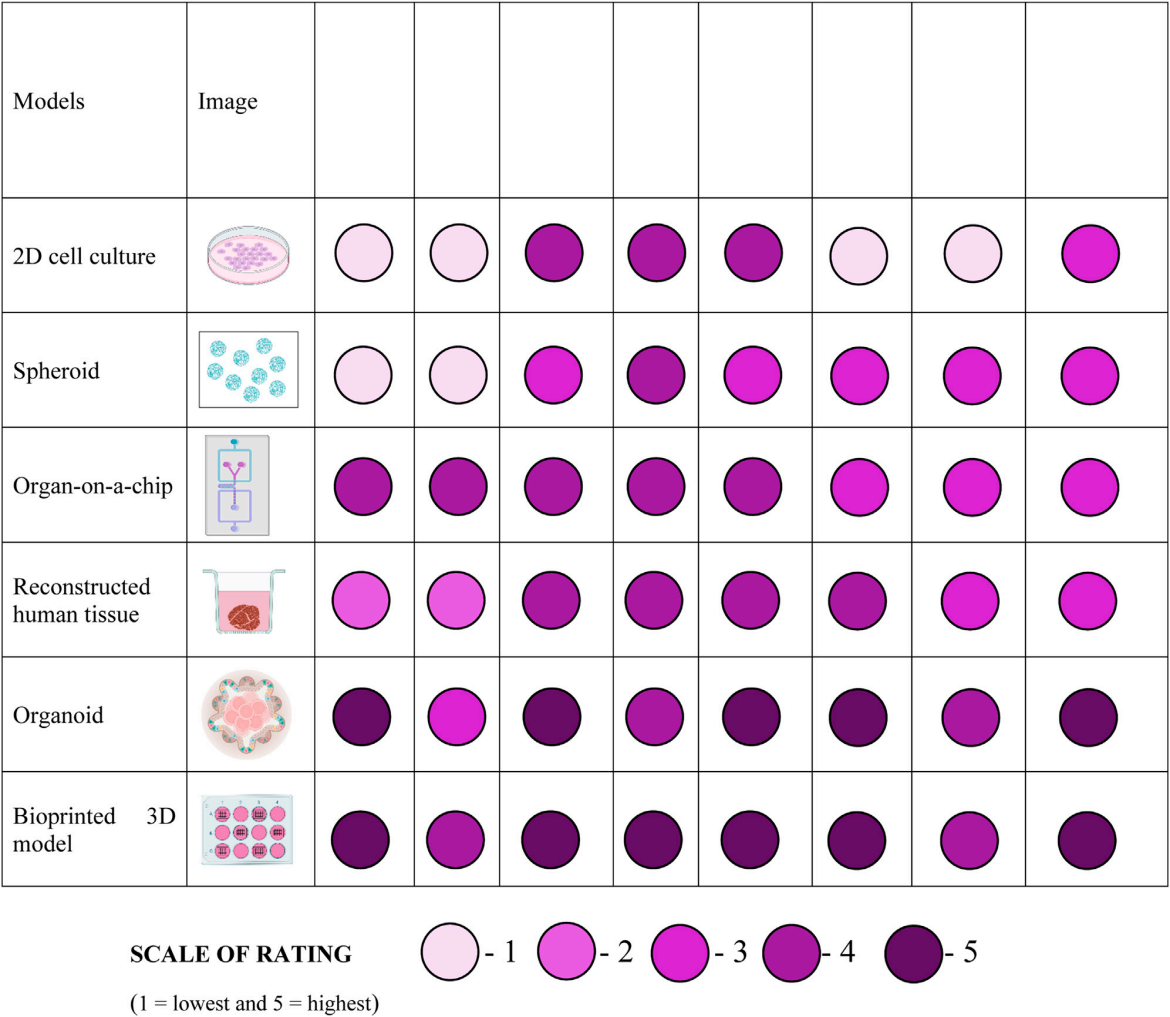

#### 3.1.1 2D cell culture

Monolayer or 2D cell culture involves growing cells on a flat surface, including petri dishes and tissue culture flasks with the appropriate medium and supplements for cell proliferation. Physiological conditions are provided, and the cells adhere to the surface and proliferate (Peng et al., 2017). 2D cell culture is an essential tool for early studies on drug absorption, distribution, metabolism, excretion, and toxicity before moving on to more complex models, particularly in anticancer drug discovery (Abolhasani et al., 2018). Some of the many recent applications of 2D cell culture in drug discovery include intestinal drug absorption with Caco-2 cells (Cai et al., 2014), drug-induced liver injury and enzyme response to medications with sandwich-cultured hepatocytes (Yang et al., 2016), and epigenetic drug testing with C2C12 cells (Ikeda et al., 2017).

This method has certain limitations that impact its usefulness for modeling physiological systems accurately, such as significant deviations in cell behavior from typical physiological behavior and alteration in polarization state (Kapałczyńska et al., 2016; Fontoura et al., 2020).

#### 3.1.2 Mini tissues

##### 3.1.2.1 Hollow fibers as 3D models

These models utilize biocompatible hollow polyvinylidene fluoride (PVDF) fibers, which allow the movement of proteins while containing the seeded tumor or bacterial cells (Nam et al., 2015). Once cultured *in vitro*, these fibers are implanted in immunodeficient mice in subcutaneous and intraperitoneal sites. Candidate drugs are then administered to the mice for a period of 6–8 days. Afterward, the fibers are removed, and colorimetric assays such as the MTT assay are employed to determine the viable cell mass and drug effectiveness.

Traditionally, hollow fiber models were used to study anticancer drug sensitivity (Casciari et al., 1994) and to screen novel antiproliferative agents for different types of cancer (Decker, et al., 2004). Srivastava et al. (2016) developed a 3D organotypic liver hollow fiber model to study the efficacy of drugs for treating tuberculosis in pediatric patients. The results indicated that the drug pyrazinamide which is commonly used for tuberculosis treatment, had reduced treatment efficacy compared to isoniazid. This study highlights the need of personalized treatment regimes for pediatric patients. Hollow fiber models are also applicable for antibiotic testing, as demonstrated by Kloprogge et al. (2019), who evaluated the efficacy of anti-tubercular drugs by generating lung lesion homogenate in a hollow fiber model, and by Pieterman et al. (2021), who studied the effects of rifamycin dosage against *Mycobacterium tuberculosis*.

Hollow fiber models are very versatile, and by lowering the number of animal models and test substances needed, help in quicker identification of effective drugs. However, this model reduces the period of cell viability and slows down the passage of glucose and oxygen and larger molecules like antibodies and gene therapy vectors (Casciari et al., 1994).

##### 3.1.2.2 Spheroids as 3D models

Spheroid generation is based on the ability of cells to undergo self-assembly and self-organization (Laschke and Menger, 2017). Compact, solid spheroids are formed by the interaction of the free cells with ECM fibers and homophilic binding of cadherin (Cui et al., 2017). Spheroids can be monocultures of a single cell type or co-cultures of various cell types to mimic the tissue microenvironment accurately.

Tumor spheroids play a crucial role in oncology research. Pickl and Ries (2009) demonstrated drug resistance in HER2-positive breast cancer by mimicking the homodimerization of HER2, and Doublier et al. (2012) identified the mechanism of doxorubicin resistance in breast cancer by using MCF7 spheroids. The suitability of Regorafenib as an adjuvant chemotherapy agent for colon cancer was also discovered by using multicellular spheroids of colon cancer HCT116 cells (Yoshii et al., 2016). Spheroids can also be employed for genetic screening, as demonstrated by Schlabach et al. (2008). The team used tumor spheroids in rotary cell culture and spinner systems to identify shRNAs with antiproliferative effects.

However, controlling the cell number and location in heterogeneous tumor spheroids is difficult. While the cells in spheroids can produce the extracellular matrix, the composition is different from that produced by native tumors, and this can alter drug response (Belfiore et al., 2021).

### 3.2 Organ-on-a-chip (OOC)

Microfluidics, biomimicry, and microengineering are combined to create OOCs. OOC systems have chambers that can accommodate living cells, and the carefully regulated fluid (medium) flow into the chambers promotes cell growth and allows the interaction of cell-produced metabolites with one another, simulating physiological conditions in the body.

Multi-organ-on-a-chip (MOOC) models consist of two or more organ chambers connected by microchannels to promote multi-organ communication (Zheng et al., 2016). The pharmacokinetic and pharmacodynamic information acquired by MOOCs is comparable to *in vivo* circumstances, and they can mimic many crucial drug metabolic pathways, including prodrug activation. These systems are applied in ADMET studies since they can incorporate the major organs involved – intestine (absorption), blood vessel system (distribution), liver (metabolism), kidney (excretion), and the target organs (for example, kidney, heart or brain for investigating nephrotoxicity, cardiotoxicity or neurotoxicity respectively) (Picollet-D’hahan et al., 2021). MOOCs are also well suited for modeling systemic diseases, including metabolic diseases, due to their cross-organ communication (Jang and Kim, 2023). By seeding the chip with cells from a single patient or patients of a given gender, it can analyze patient- and gender-specific drug interactions, bringing this method one step closer to personalized medicine (Palaninathan et al., 2018; Ingber, 2022).

One of the drawbacks of MOOCs, namely the lack of monitoring of oxygen and metabolite levels, is overcome by using integrated OOCs. In integrated OOCs, the embedded biosensors monitor the microenvironment (pH, dissolved oxygen, temperature), cell metabolism and function (biomarkers, barrier integrity), and response to external stimuli, which can be electrical, mechanical, or drugs (Zhu et al., 2021). Based on the required applications, mechanical, pressure, electrochemical, and optical sensors can be used (Chen et al., 2023). In a study, embedded electronic sensors in a cardiac OOC were used to determine the effect of 2 model drugs on the contractile forces of cardiac cells cultured on the chip (Lind et al., 2016). Similarly, Wang L. et al. (2018) developed a microdevice array with embedded carbon nanotube-based stress sensors to quantify alterations in contractility and beating rate and rhythm of human iPSC-CMs (cardiomyocytes) as a response to various drugs. Integrated OOCs can also be used for studying drug toxicity, as shown by Shin et al. (2017). The team developed a liver-on-chip model with electrochemical biosensors to monitor hepatotoxicity biomarkers secreted in response to the hepatotoxic drug acetaminophen.

### 3.3 Organoids

Organoids are self-organizing 3D models developed from cells that structurally, biologically, functionally, and developmentally mimic a specific organ of interest. They can be derived from induced pluripotent stem cells or various tissue-derived cells such as adult stem cells, differentiated cells, or cancer cells (Hofer and Lutolf, 2021). They can be used to study the developmental processes of organs, disease conditions, and drug responses. Organoids also find applications in cancer research, since they maintain genetic and phenotype stability and can be cryopreserved easily (Drost and Clevers, 2018).

A significant advantage of organoids in disease modeling and drug development is the ability to create personalized models using the patient-derived cells, which has been studied extensively. Freedman et al. (2015) developed kidney tubular organoids from human pluripotent stem cells with drug responses similar to those found in clinical settings. The model was also amenable to CRISPR/Cas9-based genome editing, which was used to induce polycystic kidney disease characteristics in the organoid Votanopoulos et al. (2019) conducted a study using patient-specific immune-enhanced tumor/node organoids for immunotherapy screening. The responses of the organoids to various immunotherapy drugs were very similar to the clinical response, showing the feasibility of these organoids for patient-specific drug testing. However, further studies are needed to fully understand and widen the applications of organoids.

### 3.4 3D bioprinting

3D bioprinting is a technique derived from additive manufacturing which involves the deposition of viable cells combined with a suitable biomaterial, referred to as bioink, in a layer-by-layer fashion to fabricate *in vitro* tissue or organ constructs (Lee et al., 2018). The various types of 3D bioprinters can be broadly classified as – inkjet-based, laser-based, and extrusion-based (Dababneh and Ozbolat, 2014). Inkjet-based bioprinting employs bioink deposition as droplets or microspheres, having advantages like low cost and high precision. However, the applications are limited mainly due to nozzle blockage during the printing of bioinks with high cell densities. In laser-based bioprinting, the bioink droplets are deposited without any contact with the substrate. Since laser-based bioprinters are nozzle-free, the drawbacks of inkjet-based bioprinters are overcome, and the generated constructs are more reproducible. However, the drawbacks include the difficulty of process adjustment and control and the limitations in developing complex constructs. In extrusion-based bioprinting, the bioink is deposited as filaments stacked on the substrate to develop the construct. It is a very versatile technique, and a wide variety of bioinks are suitable for printing, so this technique is most employed for 3D bioprinting. The drawbacks include the lower resolution in the printed constructs, which can negatively impact the replication of the tissue microenvironment, and the shear force during printing can affect the cell characteristics (He et al., 2020).

**FIGURE 2:**
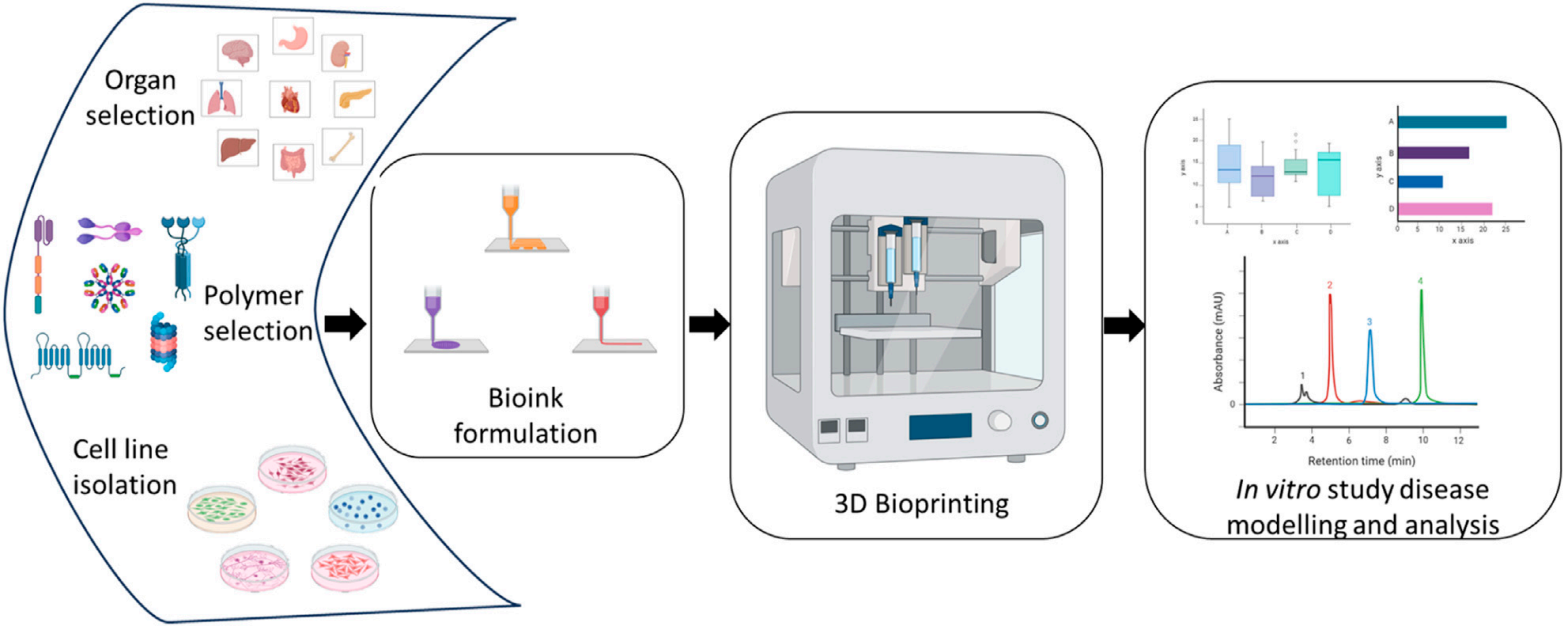
Outline of 3D bioprinting process for disease modelling and analysis.

The 3D bioprinting process can be divided into preprocessing, processing, and postprocessing steps. In the first step, the cells to be included in the bioink are grown, and the CAD model of the required construct is generated to be used as the blueprint. The bioink is developed and characterized during bioprinting, and the appropriate method of 3D bioprinting is followed. In the postprocessing phase, the construct is allowed to grow in a bioreactor and the cell interactions and viability are routinely examined (Datta et al., 2018; Levato et al., 2020). Figure 2 briefly describes the fabrication of a 3D bioprinted *in vitro* model.

3D bioprinting has extensive applications in tissue engineering, pharmaceutical sciences, biomedical sciences, and other allied fields. This review will explore the applications of 3D bioprinting in the pharmaceutical science context, particularly in drug discovery and testing and disease modelling. Further improvements in this technology can comply with the 3 Rs of drug testing – Replacement, Reduction, and Refinement. They can significantly reduce the reliance on animal studies, giving a more personalized approach to drug discovery.

### 3.5 Advantages of 3D bioprinting for drug discovery and development

3D bioprinting offers a promising alternative to traditional 2D cell culture and other 3D models. It enables the replication of the ECM and its interactions, making it a valuable tool for creating personalized disease models using patient cells (Pagnotta et al., 2022). This technique provides versatility by allowing the incorporation of drugs, genes, and growth factors into the printed models. By selecting the appropriate hydrogel, controlled drug release mechanisms can be studied (Satpathy et al., 2018). For example, Faramarzi et al. (2018) developed patient-specific alginate-based bioink incorporated with platelet-rich plasma (PRP) for cardiovascular and musculoskeletal tissue engineering. The introduction of patient-specific growth factors from PRP makes the developed bioink personalized for the patient.

During the initial stages of drug discovery, such as target selection, testing a wide range of dosages is necessary. Utilizing 3D bioprinted models allows for a high-throughput approach, yielding quick and accurate results, as demonstrated in various studies. Assessing toxicity is crucial to drug development, particularly hepatotoxicity and cardiotoxicity. Animal models often lack human-specific drug-metabolizing enzymes, but 3D bioprinted liver and heart models used in drug screening provide more accurate data on drug toxicity (Mazzocchi et al., 2019; Faulkner-Jones et al., 2015). For example, Hong and Song (2021) 3D bioprinted liver spheroids using an alginate-gelatin hydrogel encapsulated with human hepatocarcinoma cells (HepG2) and screened them against the drugs nefazodone, troglitazone and dexamethasone to monitor the drug-induced hepatotoxicity. 3D bioprinted cardiac spheroid models were used for high throughput analysis of doxorubicin toxicity by Khoury et al. (2023). The bioink used was AC16 cardiomyocytes in alginate-gelatin hydrogel, and the drug-induced cardiotoxicity on the spheroids was compared with a 2D cell culture model. The results indicated that the spheroid models were better for monitoring cardiotoxicity, however, further investigations are needed to develop a physiological model since these cells do not accurately mimic cardiac tissue behavior.

**FIGURE 3:**
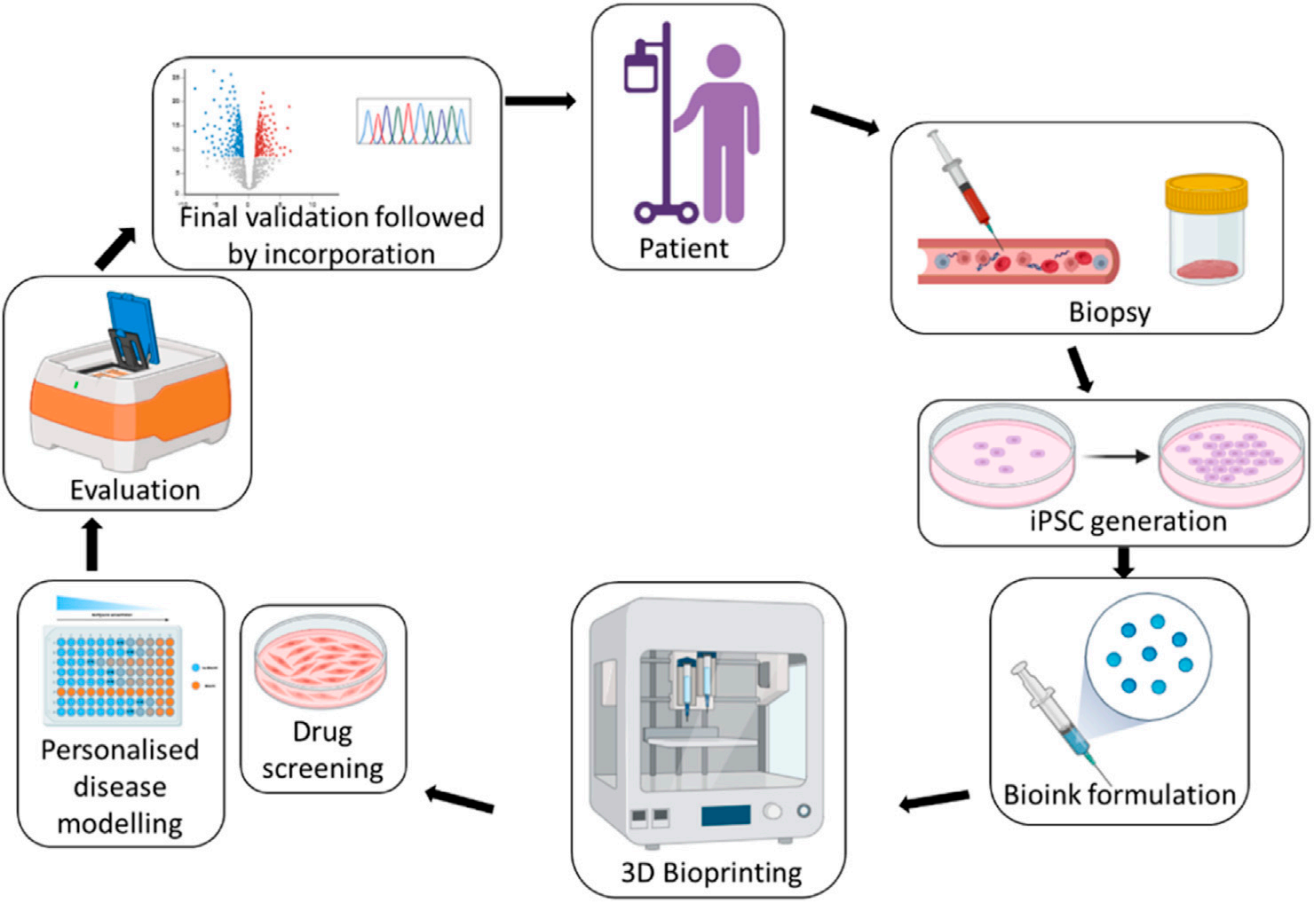
Overview of personalized biofabrication for patient-specific drug testing.

Thus, 3D bioprinted models of healthy and diseased tissues and organs are increasingly important in drug discovery and development (Kasturi et al., 2023). The following sections will explore numerous examples of such models successfully employed in drug screening research. Figure 3 represents the development of patient-specific 3D bioprinted models for the personalised drug testing studies.

## 4 3D bioprinted models for disease progression and drug screening

### 4.1 Bioinks for 3D bioprinting

In the field of tissue engineering, bioink formulation is key for creating 3D bioprinted models that accurately mimic the native tissue environments. A variety of natural, synthetic, and hybrid biopolymer materials are used for this purpose. The most used natural polymers include collagen, chitosan, alginate, and gelatin methacrylate (Yin et al., 2018; Naghieh et al., 2019; Ku et al., 2020; Yang et al., 2022). Commonly used synthetic polymers include polylactic acid (PLA), polycaprolactone (PCL), poly(lactic-co-glycolic acid) (PLGA), and polyvinyl alcohol (PVA) (Murphy et al., 2017; Wu et al., 2019; Zamani et al., 2020; Rizwana et al., 2023). Hybrid polymers are extensively employed for bioink formulation because they combine the biocompatibility of natural polymers with the favourable properties of synthetic polymers, such as good mechanical strength and tunable rate of degradation. Commonly used hybrid polymers are combinations of alginate, collagen, or gelatin with synthetic biomaterials such as PLGA, PVA, or polyethylene glycol (PEG).

In addition to using natural, synthetic, and hybrid polymers, there is growing interest in using extracellular matrix (ECM) analogues for bioink formulation. Although the ECM is derived from the native tissues, the decellularization and solubilization steps can alter its composition. However, it provides a close representation the tissue microenvironment, thus enabling studies on cell-ECM interactions that play a key role in disease pathophysiology and drug response. 3D bioprinting enables the fabrication of ECM with the specific cell types, porosity, and stiffness corresponding to the target organ or disease (Cleversey et al., 2019). Pati et al. (2014) developed decellularized extracellular matrix (dECM) bioinks from adipose, cartilage, and heart tissues for 3D bioprinting that incorporated human adipose-derived stem cells and human tissue-derived mesenchymal stem cells. The printed constructs supported cell viability and functionality and facilitated tissue-specific gene expression and differentiation. The research indicates that such bioinks can be used for developing ECM analogues for tissue reconstruction, having various applications in drug testing and disease modeling. A similar study by Yi et al. (2019) involved developing a glioblastoma (GBM)-on-a-chip model with three types of materials – bioink made of brain dECM loaded with patient-derived GBM cells, bioink consisting of human umbilical vein endothelial cells (HUVECs)-loaded brain dECM, and silicone ink. Sequential printing of these different inks accurately mimicked the native clinical environments and exhibited patient-specific resistances to standard treatments. This highlights the potential of this model to be used for predicting treatment options, an essential step towards personalized therapy.

In 2018 a study by Ma et al. used light-based 3D bioprinting to create a photocrosslinkable liver extracellular matrix to study the impact of matrix stiffness on hepatocellular carcinoma (HCC) progression and invasion. By encapsulating HepG2 cells in liver dECM scaffolds with cirrhotic stiffness, they discovered that these cells displayed decreased growth and higher levels of invasive markers in comparison to the healthy controls. This highlighted a correlation between matrix stiffness and HCC progression.

Tri-regional models of GBM representing the tumour region, acellular ECM region, and endothelial region were created via 3D bioprinting by Tang et al. (2021). Patient-derived GBM cells, hyaluronic acid derivatives, and human endothelial cells were utilized in the study. Different stiffness models were created, and varied morphologies and patterns were seen in the invasion of tumour cells into the ECM regions, indicating the influence of these stiffness conditions for modelling various GBM subtypes. While stiff models displayed hypoxia, stemness, angiogenic potential, and enrichment of gene sets linked to mesenchymal and procedural lineages corresponding to primary GBM tissues, soft models demonstrated rapid cell proliferation and enrichment of gene sets associated with the classical subtype. Furthermore, the study evaluated the effects of temozolomide (TMZ) treatment on GBM models, finding that stiff and stiff-coculture models showed higher drug resistance. This study demonstrates the significance of regional stiffness in regulating GBM cell behaviour and drug resistance, providing insights for developing more effective therapeutics for GBM treatment.

### 4.2 Established organ models

#### 4.2.1 Bone and cartilage

3D bioprinting has been applied to model various bone disorders, for instance, the 3D bioprinter parameters can be adjusted to produce bone structures that replicate the conditions of osteoporosis and Paget’s disease (Barak and Black, 2018). Zhou et al. (2016) studied breast cancer bone metastasis using 3D bioprinting, encapsulating osteoblasts and MSCs in GelMA hydrogel with nHA. When these cells were co-cultured with breast cancer (BrCa) cells, BrCa cell growth was stimulated while osteoblast or MSC proliferation was inhibited. Additionally, in the co-culture, VEGF secretion increased in BrCa cells and alkaline phosphatase activity decreased in osteoblasts and MSCs. Breathwaite et al. (2020) developed 3D bioprinted bone models for drug screening, evaluating Icariin, Purmorphamine, PD98059, and U0126 on bioprinted bone marrow-derived MSC spheroids. Icariin and Purmorphamine enhanced osteogenesis, evident through increased mineralization, ALP activity, and expression of osteogenesis-related genes. Conversely, PD98059 and U0126 treatment led to reductions in these indications. The study concluded that Icariin promoted osteogenesis more effectively, while U0126 inhibited osteogenesis more effectively.

The potential of 3D bioprinting to recreate osteochondral-like structures with distinct layers corresponding to articular/hyaline cartilage and subchondral bone has been discussed by Maia et al. (2023). This presents significant opportunities for modeling osteoarthritis pathophysiology and facilitating drug screening. Singh et al. (2022) developed an early osteoarthritis model using 3D-printed cartilage and bone layers containing encapsulated pre-differentiated chondrocytes and osteoblasts, respectively. These cells were cultivated in a pro-inflammatory culture medium supplemented with IL-1β and TNFa, simulating early osteoarthritic stages. Subsequently, the culture was treated with two anti-inflammatory drugs, Celecoxib (CXB) and Rhein (RHN). Post-treatment, osteoarthritic symptoms such as reduced expression of bone markers and elevated nitric oxide levels showed improvement, validating the model’s efficacy for screening potential osteoarthritic drugs.

#### 4.2.2 Cutaneous tissue (skin)

Skin serves as a protective barrier against external factors and is constantly exposed to various stresses. Thus, there is a huge demand for skin models for regenerative medicine and drug testing applications.

In a study, Liu et al. (2020) used 3D bioprinting and microfluidics to create a vascularized model of atopic dermatitis (AD). They induced AD in a 3D bioprinted skin model by introducing IL-4. By evaluating the model’s response to dexamethasone and Janus kinase inhibitors, they found that the observed pharmacological responses correlated with clinical data. Bin et al. (2022) developed a human hypertrophic scars (HSS) model using preformed cellular aggregates bioprinting. This model accurately represented early-stage HSS gene and protein expression and relevant signaling pathway activation. Testing two drugs, Abemaciclib and Cobimetinib, revealed that Cobimetinib exhibited superior effects compared to the clinically used drug by modulating the expression of fibrotic markers. These models can be further developed for employment in preclinical studies, allowing the evaluation of drug effectiveness and exploring disease mechanisms, ultimately leading to improved treatments for skin disorders.

#### 4.2.3 Liver

The role of the liver in drug metabolism underscores the need to develop 3D bioprinted liver models for drug discovery and disease modeling. Janani et al. (2022) investigated the toxicity of different types of drugs in a 3D bioprinted liver model, including non-hepatotoxic drugs like aspirin and dexamethasone, idiosyncratic hepatotoxic drugs like trovafloxacin mesylate, and hepatotoxic drugs like acetaminophen and troglitazone. The model effectively metabolized the drugs and accurately represented hepatotoxicity. This suggests that the 3D bioprinted liver model can be utilized for high-throughput drug screening.

The effectiveness of anticancer drugs has also been assessed using 3D bioprinted liver models. In a study by Sun et al. (2020), the anticancer efficacy of cisplatin, sorafenib, and regorafenib was compared in a bioprinted liver model. The researchers found that the model could reliably predict the effectiveness of these anticancer drugs compared to 2D models. Norona et al. (2016) investigated the relationship between drug-induced liver injury and the development of fibrosis using 3D bioprinted liver models. They induced liver damage and progressive fibrogenesis in the models by exposing them to methotrexate and thioacetamide. This led to collagen accumulation in distinct patterns, accurately simulating the stages of liver damage and fibrosis progression.

#### 4.2.4 Brain and nervous tissue

The intricacies of the human nervous system, combined with limited access to primary samples, pose significant challenges to neurological research. Animal models have limitations, and existing *in vitro* models need improvements. 3D bioprinted models offer a more representative platform for studying neurological processes, making them ideal for disease modeling and drug screening studies.

Dai et al. (2016) developed a 3D bioprinted glioma stem cell model using a porous gelatin/alginate/fibrinogen hydrogel that mimics brain tumor ECM. The cultured glioma stem cells retained their cancerous characteristics and had the potential for vascularization. When the model was treated with temozolomide (TMZ), the 3D model showcased higher resistance than the 2D models, making it more accurate and reliable for studying gliomagenesis, glioma stem cell biology and the effects of anticancer drugs. Lee et al. (2019) 3D bioprinted a fibrin-based glioblastoma multiforme (GBM) model and it was observed that the model allowed the printed cells to self-organize into spheroids, thus forming a tumor-like environment. The upregulation of key proteins associated with cancer stem cells and metastasis was also observed. When the model was treated with a known GBM-reprogramming cocktail, it was observed that the 3D model represented the *in vivo* response more accurately than 2D cultures.

Abdelrahman et al. (2022) 3D bioprinted ultrashort self-assembling tetrapeptide bioinks to create 3D models of dopaminergic (DA) neurons to mimic the conditions of Parkinson’s disease (PD). By exposing these models to the neurotoxin 6-hydroxydopamine (6-OHDA) and combining them with endothelial cells, they recreated conditions resembling neurodegeneration and vascularization found in the brain microenvironment, which is a hallmark of PD. Spontaneous action potentials of the DA neurons were recorded, validating the authenticity and physiological relevance of the 3D models. This model can investigate PD pathophysiology and help explore the effectiveness of potential therapeutic agents. Few of the other studies carried out are mentioned in Table 3.

**TABLE 3:** Different studies used as *in vitro* drug testing models.

Models	Sl. No.	Cell lines used	Study tested	Model developed	Study specifics	References
2D cell culture	1	cancer cell lines (MDA-MB-231, MCF-7, Hep-G2, and A-549)	Toxicology study	(disease model)	Four different cancerous cell lines were used to study cytotoxicity level induced by various algal extracts. Apoptosis in these models were analysed by different methods like AO/EB and V-FITC/PI staining, assays like caspase-3, ROS measurement and MMP.	Abolhasani, et al. (2018)
	2	Caco-2 and MDCK	Drug study	(disease model)	Using two different cell lines they evaluated by two methods liquid chromatography and mass spectrometry; this study measures the permeability coefficient (Papp) to evaluate the anti-malarial drug	Jin X et al. (2014)
	3	primary rat hepatocytes	Drug study	(disease model)	This model was established to optimize the cytochrome (CYP450) assay in respect with time point to exposure as well the duration of the three drugs α-naphthoflavone (ANF), β-naphthoflavone (BNF) and trans-resveratrol (RES). All these drugs are well known for stimulation and inhibition effect on CYP450 enzymes	Schaeffner et al. (2005)
	4	Caco-2	Drug study	(disease model)	The study produces a novel system in providing a rapid and economical option that can assess permeability of the drug and this also can be applied in the study of understanding the absorption of drug in the intestines	Cai Y et al. (2014)
	5	Human hepatocytes sandwich-cultured with MPA and MPAG	Toxicology studies	(disease model)	This study has developed a human hepatocyte sandwich culture produced by mathematical modelling for evaluating the interaction of CsA with hepatic disposition	Matsunaga et al. (2014)
Spheroids	1		Drug study	(disease model)	This study developed a system termed as microwell-mesh that enabled the manufacturing of almost 150 microtumours per well in a 48 well plate which provides platform for studing the response of anti-tumour drugs	Mosaad et al. (2018)
	2		Toxicology study	Cancer (disease model)	This study developed a spheroid as a multicellular tumour model to identify new targets in order to treat HER2-positive patients having breast cancer	Pickl and Ries (2009)
	3	MCF7	Drug study	(disease model)	This study investigated the activation of hypoxia-inducible factor-1 (HIF-1) leading to the resistance of doxorubicin used in treatment of tumour in a 3D model composed up of cancer cells	Doublier et al. (2012)
	4	MIAPaCa-2 and PANC-1	Drug study	(disease model)	This study developed a spheroid-based 3D culture for mimicking pancreatic cancer for testing drugs on pancreas. The drug testing efficiency was better and much higher than the traditional 2D monolayer cells	Wen et al. (2013)
	5	PDAC	Toxicology study	(disease model)	This study developed a basic yet high throughput 3D model to explore the transition from 2D to 3D which might be responsible for the chemoresistance	Longati et al. (2013)
Organ-on-a-chip	1	hPSC-CMs	Drug study	Heart (disease model)	This study developed an engineered heart that is clinically relevant tissues that composed of chamber specific cells. This model used the EHTs that was generated by directing hPSCs differentiation to cardiomyocytes which were embedded into the collagen hydrogel that generated chamber specific and ring shaped models	Goldfracht et al. (2020)
	2	hPDAC	Toxicology studies	(disease model)	This model was used to characterize the development of hPDAC microenvironment tissue. They have used InVADE platform for 3D vascularized tumor tissue derived from patient. They have used organoid technology that was combined with bio-scaffolds that was able to mimic vessel that was perfusable and vascularized	Lai et al. (2020)
	3	Cardiomyocytes	Drug study	Heart (disease model)	They generated a heart-on-chip and established a model that supports different functions like metabolism, heart contraction and the cellular viability upon injury induction	Yadid et al. (2020)
	4	bronchial epithelia and pulmonary endothelia	Toxicology studies	(disease model)	The study developed a bronchia-on-a-chip, that can be used for various studies like Model for viral infections, production of cytokines and recruitment for immune cellsfrom the circulation.	Longlong et al. (2021)
	5	sinusoidal endothelial cells, kupffer cells and hepatic stellate cells	Toxicology study	Liver (disease model)	The study developed a micro-engineered liver-on-chip that could be used on a rat, dog and human. The cultured cells were supplemented by using the method of flowing physiological fluid for the model to mimic liver-like nature. They claim that the model could detect diverse phenotypes including toxicity of liver, liver injury such as fibrosis, cholestasis when treated with different compounds	Jang et al. (2019)
	6			Intestine (disease model)	A four-organ micro-physiological model that could mimic the functions over a period of 28 days in a co-culture environment	Maschmeyer et al. (2015)
	7		Drug study	(disease model)	This study has described three differently designed mixing chambers, 4-, 7- and 10-way MPSs. The system has different MPSs that are physiologically linked together by a microfluidic system, this makes them compatible to perform quantitative study for different compounds. The model performs enhance exchanges on molecular levels and also have a feature of pumps that are driven by pumps. The model is also well established for studying the partitioning of inter-MPSs and also distribution of drugs	Edington et al. (2018)
Bioprinted 3D models	1	Bone	Drug study	(disease model)	A 3D bioprinted model of trabecular bone has been developed for bone resorption post stimulation. They claim that this particular model can be used as a valuable tool for quantitative analysis of deterioration of mechanical property. It can be applied for the analysis by risk assessment of fracture by CT scans	Barak and Black (2018)
	2	breast cancer (BrCa) cells and bone stromal cells (fetal osteoblasts and human bone marrow mesenchymal stem cells (MSCs)	Toxicology studies	Bone (disease model)	They developed a 3D bioprinted a bone matrix that is capable of biomimicking, this model was is a technology which is applicable for interaction of different cells. The model was developed by using GelMA and nHA, with the mixture of cells. The study has demonstrated artificial microenvironment that is found in a bone matrix	Zhou X et al. (2016)
	3	differentiated bone marrow-derived mesenchymal stem cell (BM-MSC	Drug study	Bone (disease model)	This study was carried out to understand the drug interactions, one with the function of impairing osteogenic differentiations and the other with promoting osteogenesis. The study provided utility of 3D bioprinted model that was scaffold-free as an *in vitro* model for screening multiple drugs	Breathwaite et al. (2020)
	4	pre-differentiated stem cells	Drug study	Cartilage cancer (Disease model)	The 3D bioprinted model has a potential application for screening of anti-inflammatory drugs for osteochondral *in vitro* model. The model has been fabricated using silk as base material along with other stimulating agents for developing necrosis model	Singh Y et al. (2022)
	5		Drug study	Liver (disease model)	The developed liver *in vitro* model has been claimed to study the progression of HCC and was able to recapitulate the mechanical propertied of liver cirrhosis clinically. The highlight of the study is that the they have used the dECM extracted from liver that is photocross-linked in the scaffolds	Ma et al. (2018)
	6		Toxicology studies	Cancer (Disease model)	The study has demonstrated that the model has different biophysical cues that are involved in expressing tumour like property. The resistance of drug like temozolomide was studied on the model and showed high resistance	Tang et al. (2021)
	7	Patient-derived fibroblasts cells	Drug study	Cutaneous tissue (regeneration model)	They have developed a 3D bioprinted model for cutaneous tissue that uses Alginate gel and dECM from scar to make hydrogel hence making it patient specific. The model was able to successfully mimic the factors involved in the microenvironment due to involvement of the fibroblasts present in the bioink. This study has promising results for studying hypertrophic scars as well as capable as regeneration model	Bin et al. (2022)
	8	human adipose mesenchymal stem cell-derived hepatocyte-like cells (HLCs), human umbilical vein endothelial cells (HUVECs), and human hepatic stellate cells (HHSCs)	Toxicology studies	Liver (Disease model)	They claim to fabricate a liver model having the characteristic of vascularization and physiological characteristics that are relevant to the human liver microenvironment. The use of novel liver dECM based bioink involving the cells specific to liver. The model has been verified using varies assessment for hepatotoxicity and was used for studying the drug activities (aspirin dexamethasone, idiosyncratic, trovafloxacin mesylate and acetaminophen and troglitazone.) on different concentration and different time point	Janani et al. (2022)
	9	HepG2	Pharmacodynamics study	Liver (disease model)	The study developed a 3D model that involves the use of different liver specific cells to study the activity of liver biologically. The study was carried out to establish that the upcoming technology of tissue engineering that is 3D bioprinted products are way more advanced than the traditional 2D cell culture studies for understanding the pharmacodynamics	Sun et al. (2020)
	10	Glioma stem cell		Brain (disease model)	They have claimed to establish a 3D bioprinted brain model using gelatin, alginate and fibrinogen hydrogel that is capable of mimicking the brain extra cellular matrix. The model showed high cellular viability along with good cellular activity and proliferation. The model also exhibited inherent characteristics of the cells used along with differentiation potential	Dai et al. (2016)
	11	Glioblastoma	Drug study	Brain (disease model)	They developed a GBM model and used fibrin as the base for fabricating the 3D bioprinted scaffold. They have studied screening of drugs on the replicated neural tissue model. This model has proved to be having good cellular viability with increased cell proliferation and the ability to express similar microenvironmental structure as that of brain over the period of 12 months	Lee et al. (2019)
	12	mouse embryonic DA neurons	Drug study	Nervous tissue (disease model)	They fabricated a 3D bioprinted model that has used an added methodology of self-assembling of tetrapeptides in the scaffolds. The model was proven compatible with the two primary cells, both differentiated from mouse and human stem cells. The scaffold was found to be reactive to drug (6-hydroxydopamine) that was encapsulated inside the scaffold peptide. This model is proven to be successfully capable of having good signals released over a period of 1 month	Abdelrahman et al. (2022)

### 4.3 Current challenges

#### 4.3.1 Bioink selection and printing parameters

The end goal of 3D bioprinting revolves around producing fully functional organs. To achieve this, the chosen bioink or biomaterial must possess biocompatibility and mechanical properties similar to those of the natural organ. Moreover, this material should transition from a liquid state during the printing phase to a solid form in subsequent processing stages (Zhang et al., 2018). Other key aspects include degradation without releasing cytotoxic products, and vascularization. Hybrid polymers, which combine the desirable attributes of natural and synthetic polymers, are commonly employed to enhance biocompatibility. Crosslinking agents are employed to increase the mechanical strength of the 3D bioprinted constructs. However, there is still a lot to be discovered regarding the ideal materials and crosslinking agents for various 3D bioprinting applications. There are studies exploring the implementation of step-growth reactions and *in situ* photo-crosslinking for enhanced mechanical properties (Levato et al., 2020). Few of the other regulations involve the reproducibility issues, where there is a lack in achieving consistent results across different batches of production of 3D bioprinted scaffolds, which can cause variability in drug response and eventually the efficacy. Another limitation is the availability of the suitable materials and inks that can completely mimic the complex human tissue that can affect the physiological relevance during production. There are regulatory concerns that can also be a limitation and a huge drawback to the 3D bioprinted products as the products might include biological component provided by patients. Since 3D bioprinting is done in a layer-by-layer method, there has to be sufficient resolution and control over the thickness of each layer. A promising approach to address this challenge involves utilizing bioprinters with multiple printing heads. They can deposit many types of bioink simultaneously, enabling the creation of constructs with greater mechanical strength and better control over the spatial distribution of biochemical cues. Furthermore, these multi-head bioprinters can be employed during printing to facilitate organ vascularization (Chimene et al., 2016). Printing techniques such as microfluidic printing also enable the bioprinting of various materials and cell types in a single construct (Levato et al., 2020).

#### 4.3.2 Regulatory and ethical issues

3D bioprinted organs designed for transplantation must adhere to the guidelines of Good Manufacturing Practice, and their commercialization requires authorization from relevant regulatory bodies (Jessop et al., 2017). However, the existing global legal framework, designed for conventional biomedical products, is not entirely suitable for the unique characteristics of 3D bioprinted products (Chimene et al., 2016). There are additional issues to consider regarding the commercialization of 3D bioprinted organs. Customized biofabricated organs can be produced through contractual agreements involving patients and manufacturing bodies. At the same time, sale-purchase arrangements may be established for depersonalized bioprinted organs for other applications, such as drug testing (Kirillova et al., 2020). A significant ethical challenge associated with using 3D bioprinted organs is finding a balance between the advantages of personalized organs and the known and unknown transplantation risks, particularly in paediatric patients. Presently, the affordability of 3D bioprinted organ therapies remains limited, potentially widening the disparity in access to such treatments. The acceptance of 3D bioprinted organs is also heavily influenced by societal and cultural perspectives. Consequently, efforts should be directed toward public education to highlight the scientific advancements that have led to 3D bioprinted organ creation, along with their many benefits and applications (Datta et al., 2022).

The area of 3D bioprinting for drug testing has advanced rapidly, providing novel possibilities for more accurate preclinical evaluations. Regulatory issues are critical in ensuring that these technologies satisfy high safety, effectiveness, and ethical standards. As 3D bioprinting advances, regulatory organizations such as the United States Food and Drug Administration (FDA), the European Medicines Agency (EMA), and others throughout the world are actively working with industry stakeholders to provide specific regulations (Bhasale et al., 2020). The biocompatibility and safety of the materials used in 3D bioprinting are key regulatory issues. Regulatory bodies demand extensive testing of bioinks, scaffolds, and other components to guarantee they do not harm live tissues (Gilbert et al., 2018). Standardized testing for cytotoxicity, immunogenicity, and long-term safety is critical, with rules evolving to accommodate the particular issues presented by bioprinting materials (Mansouri et al., 2024). Regulatory approval is contingent on 3D bioprinted models’ capacity to consistently anticipate human medication reactions. It is critical to conduct rigorous validation studies that compare bioprinted model findings to clinical results (Ali et al., 2024). Establishing predictive skills for medication effectiveness and toxicity is critical to achieving regulatory acceptability (Barua et al., 2024). Collaboration between business, academia, and regulatory bodies is required to develop standardized validation techniques that may be applied in a variety of scenarios. Ensuring the repeatability and dependability of 3D bioprinted models is a major regulatory issue. The available standardized techniques for validation will help in assessing the safety and the efficacy of the 3D bioprinted constructs. The collaboration will also enhance the rate of generation of robust clinical data that will enable stakeholders in navigating the challenges of 3D bioprinting into mainstream medical practices. Unlike the conventional manufacturing, 3D bioprinting involves the integration of living cells that leads to different layers of variability as well as unpredictability during the production. The complexity of the constructs calls for the need of standard validations techniques. The batch-to-batch production need to ensure the consistency of the products along with the safety and effectiveness of the 3D bioprinted constructs that is applied for various applications (Gao et al., 2021; Satpathy et al., 2018). Setting up quality control methods and defined processes for the bioprinting process is critical. Parameters such as printing resolution, cell viability, and structural integrity must be specified and followed in order to create strong and consistent bioprinted constructions. Beyond technological problems, regulatory matters in 3D bioprinting include ethical concerns and public perceptions. Regulatory organizations consider ethical issues such as the use of human cells, privacy problems, and the possibility of constructing sophisticated biological structures (Pothysvaran et al., 2024). Open communication and openness are critical for addressing public concerns and fostering trust in the ethical use of 3D bioprinting technology. The emergence of individualized medicine poses new problems to regulatory structures. 3D bioprinting allows for the production of patient-specific drug testing models, necessitating a regulatory framework that supports tailored technique (Ramesh et al., 2024). Achieving a balance between personalizing medicines to specific patients and maintaining regulatory control is an important part of future regulatory advancements. Regulatory issues go beyond the pre-market period, stressing the value of real-world evidence and post-market surveillance. Continuous monitoring of the performance and safety of 3D bioprinted goods in clinical settings helps to inform continuous regulatory evaluation and adaption (Pothysvaran et al., 2024). As researchers investigate novel biomaterials for bioprinting, regulatory authorities must alter current frameworks to accommodate these breakthroughs. The approval procedure must remain adaptable in order to integrate developing materials while ensuring they fulfill safety and effectiveness requirements. Achieving worldwide regulatory norm harmonization is a critical long-term aim. Few of the regulatory aspects such as ethical issues regarding the development of 3D bioprinted scaffolds for drug screening that researchers need to keep in mind include: A. Use of cells: Incorporation of viable cells to make 3D bioprinted scaffolds concerns the ethical treatment of the models as there can be violation of consent and exploitation. B. Consent from donors: The biological source that are extracted from the donors shall have been provided with the consent and the detailed information regarding hoe the sample will be used, this applies more when the product is applicable for the commercial purposes. C. Ethics for clinical trial: The nature of 3D bioprinted models complicates the traditional trial as it is unique in every case, especially when the goal of making 3D bioprinted models for drug screening is specific in case of individual patients. D. Oversight of regulations: The advancement in the development of 3D bioprinting technology as well as the products is so rapid that it often leads to the outpacing of existing framework for regulation. There are high chances of uncertainties in case of safety, efficacy and ethical implication of such 3D bioprinted products for drug development. E. Concerns regarding commercialization: The 3D bioprinted scaffolds when commercialized raises lot of ethical questions that includes the access, equity and the profit driven motives that leads to out shadowing of patients and integrity of science. In near future there is a high demand in establishing of robust ethical guidelines for navigating the complexities involved in 3D bioprinted products for drug screening and development (Kirillova et al., 2020; Aziz, 2023).

Collaboration among regulatory bodies worldwide is critical for establishing consistent criteria that promote the international acceptance of 3D bioprinting technology for drug testing (Pothysvaran et al., 2024). Collaboration between industry stakeholders and regulatory authorities is critical for managing the developing regulatory landscape. Early involvement with regulatory authorities enables a proactive approach to compliance, which streamlines the approval process for 3D bioprinting applications. As bioprinting technology progresses to create more complicated tissue structures and organoids, regulatory hurdles become more severe. Ensuring the safety and operation of complex biological constructions demands regulatory changes to accommodate these intricacies. Considering the experimental nature of 3D bioprinting research and development, regulatory flexibility is critical for early-stage technology. Establishing channels that promote innovation while preserving safety requirements is a continuous regulatory consideration (Benson et al., 2024). The use of digital technologies in bioprinting raises issues about cybersecurity. Protecting sensitive biological data and maintaining the integrity of digital design files are two developing concerns that authorities must address in the context of 3D bioprinting. Regulatory compliance in 3D bioprinting necessitates a knowledgeable workforce. Providing education and training programs on regulatory standards ensures that professionals in the sector are prepared to deal with the complexity of compliance. Looking forward, regulatory affairs in 3D bioprinting will evolve in reaction to technical advancements. Regulatory agencies must stay adaptable, creating an atmosphere that promotes innovation while protecting public health and ethical concerns in the use of 3D bioprinting for drug testing. Continuous communication among regulators, industry, and the scientific community is critical for navigating these changing regulatory landscapes (Nel and Rymajdo, 2024).

## 5 Discussion

The integration of 3D bioprinting into pharmaceutical research has brought forth numerous options with benefits. It has paved the way for the development of more accurate, ethical, and efficient models for drug discovery and 3D disease modeling. Since 3D bioprinting is highly replicable with high throughput, drug screening is possible by using 3D *in vitro* bioartificial organs. As highlighted in this review, 3D bioprinting is highly versatile and offers a wide range of applications in developing various models for pharmaceutical studies, including complex vascularized models and disease-specific models. While certain challenges exist, such as optimizing the printing techniques, the establishment of standardization, managing mass production, and raising awareness among the public, there are significant efforts and collaborations being made to overcome these obstacles. It is crucial to involve all necessary stakeholders so that there are open and unbiased discussions about the current progress and future of 3D bioprinting (Datta et al., 2022) so that this promising technology can be fully harnessed to advance pharmaceutical research. Hence, 3D bioprinting is the gamechanger technology in the field of drug screening applications with great emphasis on reducing the use of animal models.

## References

[B1] Aban I. B. George B. (2015). Statistical considerations for preclinical studies. Exp. Neurol. 270, 82–87. 10.1016/j.expneurol.2015.02.024 25725352 PMC4466166

[B2] Abdelrahman S. Alsanie W. F. Khan Z. N. Albalawi H. I. Felimban R. I. Moretti M. (2022). A Parkinson’s disease model composed of 3D bioprinted dopaminergic neurons within a biomimetic peptide scaffold. Biofabrication 14, 044103. 10.1088/1758-5090/ac7eec 35793642

[B3] Abolhasani M. M. Safavi M. Goodarzi M. T. Kassaee S. M. Azin M. (2018). Identification and anti-cancer activity in 2D and 3D cell culture evaluation of an Iranian isolated marine microalgae *Picochlorum* sp. RCC486. DARU J. Pharm. Sci. 26, 105–116. 10.1007/s40199-018-0213-5 PMC6279668 30242672

[B4] Alcaraz J. Otero J. Jorba I. Navajas D. (2017). Bidirectional mechanobiology between cells and their local extracellular matrix probed by atomic force microscopy. Semin. Cell Dev. Biol. 73, 71–81. 10.1016/j.semcdb.2017.07.020 28743639

[B5] Ali A. S. Wu D. Bannach-Brown A. Dhamrait D. Berg J. Tolksdorf B. (2024). 3D bioprinting of liver models: a systematic scoping review of methods, bioinks, and reporting quality. Mater Today Bio 26 (100991), 100991. 10.1016/j.mtbio.2024.100991 PMC10978534 38558773

[B6] Arrowsmith J. Miller P. (2013). Trial watch: phase II and phase III attrition rates 2011-2012. Nat. Rev. Drug Discov. 12, 569. 10.1038/nrd4090 23903212

[B7] Aziz M. F. B. A. (2023). “Mapping the ethical and regulatory issues of 3D bioprinting using biomaterials in a low-and middle-income nation: Malaysian perspectives,” in Sustainable material for biomedical engineering application (Singapore: Springer Nature Singapore), 467–482. 10.1007/978-981-99-2267-3_22

[B8] Barak M. M. Black M. A. (2018). A novel use of 3D printing model demonstrates the effects of deteriorated trabecular bone structure on bone stiffness and strength. J. Mech. Behav. Biomed. Mater. 78, 455–464. 10.1016/j.jmbbm.2017.12.010 29241149 PMC5758409

[B152] Bade R. Chan H. Reynisson J. (2010). Characteristics of known drug space. Natural products, their derivatives andsynthetic drugs. European. J. Medici. Chemis. 45 (12), 5646–5652. 10.1016/j.ejmech.2010.09.018 20888084

[B9] Barua R. Das D. Biswas N. (2024). “Revolutionizing drug discovery with artificial intelligence: enhancing efficiency, addressing ethical concerns, and overcoming limitations,” in Approaches to human-centered AI in healthcare. Editors Grover V. Baluswamy B. M. K. N. Anand V. Milanova M. (IGN Global).

[B10] Belfiore L. Aghaei B. Law A. M. K. Dobrowolski J. C. Raftery L. J. Tjandra A. D. (2021). Generation and analysis of 3D cell culture models for drug discovery. Eur. J. Pharm. Sci. 163, 105876. 10.1016/j.ejps.2021.105876 33989755

[B11] Benson C. Obasi I. C. Akinwande D. V. Ile C. (2024). The impact of interventions on health, safety and environment in the process industry. Heliyon 10, e23604. 10.1016/j.heliyon.2023.e23604 38173504 PMC10761781

[B12] Bhasale A. L. Sarpatwari A. De Bruin M. L. Lexchin J. Lopert R. Bahri P. (2020). Postmarket safety communication for protection of public health: a comparison of regulatory policy in Australia, Canada, the European Union, and the United States. Clin. Pharmacol. Ther. 109, 1424–1442. 10.1002/cpt.2010 32767557

[B13] Bin Y. Zhu D. Cui X. Enhe J. Wei S. Zhao L. (2022). Modeling human hypertrophic scars with 3D preformed cellular aggregates bioprinting. Bioact. Mater 10, 247–254. 10.1016/j.bioactmat.2021.09.004 34901543 PMC8636708

[B14] Breathwaite E. Weaver J. Odanga J. dela Pena-Ponce M. Lee J. B. (2020). 3D bioprinted osteogenic tissue models for *in vitro* drug screening. Molecules 25, 3442. 10.3390/molecules25153442 32751124 PMC7435717

[B15] Bunton D. (2011). The use of functional human tissues in drug development. Cell Tissue Bank. 12, 31–32. 10.1007/s10561-010-9213-5 20824349

[B16] Cai Y. Xu C. Chen P. Hu J. Hu R. Huang M. (2014). Development, validation, and application of a novel 7-day Caco-2 cell culture system. J. Pharmacol. Toxicol. Methods 70, 175–181. 10.1016/j.vascn.2014.07.001 25034865

[B17] Cardoso B. D. Castanheira E. M. Lanceros‐Méndez S. Cardoso V. F. (2023). Recent advances on cell culture platforms for *in vitro* drug screening and cell therapies: from conventional to microfluidic strategies. Adv. Healthc. Mater. 12 (18), 2202936. 10.1002/adhm.202202936 36898671 PMC11468737

[B18] Casciari J. J. Hollingshead M. G. Alley M. C. Mayo J. G. Malspeis L. Miyauchi S. (1994). Growth and chemotherapeutic response of cells in a hollow-fiber *in vitro* solid tumor model. J. Natl. Cancer Inst. 86, 1846–1852. 10.1093/jnci/86.24.1846 7990159

[B19] Chávez-Hernández A. L. López-López E. Medina-Franco J. L. (2023). Yin-yang in drug discovery: rethinking *de novo* design and development of predictive models. Front. Drug Discov. 3, 1222655. 10.3389/fddsv.2023.1222655

[B20] Chen H. Luo Z. Lin X. Zhum Y. Zhao Y. (2023). Sensors-integrated organ-on-a-chip for biomedical applications. Nano Res. 16, 10072–10099. 10.1007/s12274-023-5651-9 PMC10130312 37359077

[B21] Chimene D. Lennox K. K. Kaunas R. R. Gaharwar A. K. (2016). Advanced bioinks for 3D printing: a materials science perspective. Ann. Biomed. Eng. 44, 2090–2102. 10.1007/s10439-016-1638-y 27184494

[B22] Cleversey C. Robinson M. Willerth S. M. (2019). 3D printing breast tissue models: a review of past work and directions for future work. Micromachines 10, 501. 10.3390/mi10080501 31357657 PMC6723606

[B23] Cui X. Hartanto Y. Zhang H. (2017). Advances in multicellular spheroids formation. J. R. Soc. Interface 14, 20160877. 10.1098/rsif.2016.0877 28202590 PMC5332573

[B24] Dababneh A. B. Ozbolat I. T. (2014). Bioprinting technology: a current state-of-the-art review. J. Manuf. Sci. Eng. 136, 061016. 10.1115/1.4028512

[B25] Dai X. Ma C. Lan Q. Xu T. (2016). 3D bioprinted glioma stem cells for brain tumor model and applications of drug susceptibility. Biofabrication 8, 045005. 10.1088/1758-5090/8/4/045005 27725343

[B26] Datta P. Barui A. Wu Y. Ozbolat V. Moncal K. K. Ozbolat I. T. (2018). Essential steps in bioprinting: from pre-to post-bioprinting. Biotechnol. Adv. 36, 1481–1504. 10.1016/j.biotechadv.2018.06.003 29909085

[B27] Datta P. Cabrera L. Y. Ozbolat I. T. (2022). Ethical challenges with 3D bioprinted tissues and organs. Trends Biotechnol. 41, 6–9. 10.1016/j.tibtech.2022.08.012 36117024

[B28] Decker S. Hollingshead M. Bonomi C. A. Carter J. P. Sausville E. A. (2004). The hollow fibre model in cancer drug screening. Eur. J. Cancer 40, 821–826. 10.1016/j.ejca.2003.11.029 15120037

[B29] Dias D. A. Urban S. Roessner U. (2012). A historical overview of natural products in drug discovery. Metabolites 2, 303–336. 10.3390/metabo2020303 24957513 PMC3901206

[B30] Doublier S. Belisario D. C. Polimeni M. Annaratone L. Riganti C. Allia E. (2012). HIF-1 activation induces doxorubicin resistance in MCF7 3-D spheroids via P-glycoprotein expression: a potential model of the chemo-resistance of invasive micropapillary carcinoma of the breast. BMC Cancer 12, 4. 10.1186/1471-2407-12-4 22217342 PMC3262753

[B31] Drost J. Clevers H. (2018). Organoids in cancer research. Nat. Rev. Cancer 18, 407–418. 10.1038/s41568-018-0007-6 29692415

[B32] Duan Y. M. Jin Y. Guo M. L. Duan L. X. Wang J. G. (2018). Differentially expressed genes of HepG2 cells treated with gecko polypeptide mixture. J. Cancer 9 (15), 2723–2733. 10.7150/jca.26339 30087713 PMC6072819

[B33] Edington C. D. Chen W. L. K. Geishecker E. Kassis T. Soenksen L. R. Bhushan B. M. (2018). Interconnected microphysiological systems for quantitative biology and pharmacology studies. Sci. Rep. 8, 4530. 10.1038/s41598-018-22749-0 29540740 PMC5852083

[B34] Esser T. U. Anspach A. Muenzebrock K. A. Kah D. Schrüfer S. Schenk J. (2023). Direct 3D-bioprinting of hiPSC-derived cardiomyocytes to generate functional cardiac tissues. Adv. Mater. Deerf. Beach, Fla. 35 (52), e2305911. 10.1002/adma.202305911 37655652

[B35] Faramarzi N. Yazdi I. K. Nabavinia M. Gemma A. Fanelli A. Caizzone A. (2018). Patient-specific bioinks for 3D bioprinting of tissue engineering scaffolds. Adv. Healthc. Mater 7, 1701347. 10.1002/adhm.201701347 PMC6422175 29663706

[B36] Faulkner-Jones A. Fyfe C. Cornelissen D. J. Gardner J. King J. Courtney A. (2015). Bioprinting of human pluripotent stem cells and their directed differentiation into hepatocyte-like cells for the generation of mini-livers in 3D. Biofabrication 7 (4), 044102. 10.1088/1758-5090/7/4/044102 26486521

[B37] Fontoura J. C. Viezzer C. dos Santos F. G. Ligabue R. A. Weinlich R. Puga R. D. (2020). Comparison of 2D and 3D cell culture models for cell growth, gene expression and drug resistance. Mater Sci. Eng. C 107, 110264. 10.1016/j.msec.2019.110264 31761183

[B38] Foster W. Putos S. (2014). Neglecting the null: the pitfalls of underreporting negative results in preclinical research. Available at: http://hdl.handle.net/10393/31044. 10.18192/uojm.v4i1.1036

[B39] Freedman B. S. Brooks C. R. Lam A. Q. Fu H. Morizane R. Agrawal V. (2015). Modelling kidney disease with CRISPR-mutant kidney organoids derived from human pluripotent epiblast spheroids. Nat. Commun. 6, 8715–8813. 10.1038/ncomms9715 26493500 PMC4620584

[B40] Frommlet F. Heinze G. (2021). Experimental replications in animal trials. Lab. Anim. 55 (1), 65–75. 10.1177/0023677220907617 32138592 PMC7917573

[B41] Gao G. Ahn M. Cho W. W. Kim B. S. Cho D. W. (2021). 3D printing of pharmaceutical application: drug screening and drug delivery. Pharmaceutics 13 (9), 1373. 10.3390/pharmaceutics13091373 34575448 PMC8465948

[B42] Gilbert F. O’Connell C. D. Mladenovska T. Dodds S. (2018). Print me an organ? Ethical and regulatory issues emerging from 3D bioprinting in medicine. Sci. Eng. Ethics 24, 73–91. 10.1007/s11948-017-9874-6 28185142

[B43] Giraud S. (2024). Identification of first active compounds in drug discovery. how to proceed? Front. Drug Discov. 4, 1342866. 10.3389/fddsv.2024.1342866

[B44] Goldfracht I. Protze S. Shiti A. Setter N. Gruber A. Shaheen N. (2020). Generating ring-shaped engineered heart tissues from ventricular and atrial human pluripotent stem cell-derived cardiomyocytes. Nat. Commun. 11, 75. 10.1038/s41467-019-13868-x 31911598 PMC6946709

[B45] Hagenbuchner J. Nothdurfter D. Ausserlechner M. J. (2021). 3D bioprinting: novel approaches for engineering complex human tissue equivalents and drug testing. Essays Biochem. 65, 417–427. 10.1042/ebc20200153 34328185 PMC8365325

[B46] Hartung T. (2024). The (misleading) role of animal models in drug development. Front. Drug Discov. 4, 1355044. 10.3389/fddsv.2024.1355044

[B47] Harvey W. (2001) On the motion of the heart and blood in animals. New York: The Harvard Classics.

[B48] He Y. Gu Z. Xie M. Fu J. Lin H. (2020). Why choose 3D bioprinting? Part II: methods and bioprinters. Bio-Des Manuf. 3, 1–4. 10.1007/s42242-020-00064-w

[B49] Hofer M. Lutolf M. P. (2021). Engineering organoids. Nat. Rev. Mater 6, 402–420. 10.1038/s41578-021-00279-y 33623712 PMC7893133

[B50] Hong S. Song J. M. (2021). A 3D cell printing-fabricated HepG2 liver spheroid model for high-content *in situ* quantification of drug-induced liver toxicity. Biomater. Sci. 9, 5939–5950. 10.1039/d1bm00749a 34318795

[B51] Horman S. R. (2016). “Complex high-content phenotypic screening,” in Special topics in drug discovery. Editors Chen T. Chai S. (InTech). 10.5772/65387

[B53] Hynes R. O. (2014). Stretching the boundaries of extracellular matrix research. Nat. Rev. Mol. Cell Biol. 15, 761–763. 10.1038/nrm3908 25574535

[B54] Ikeda K. Ito A. Imada R. Sato M. Kawabe Y. Kamihira M. (2017). *In vitro* drug testing based on contractile activity of C2C12 cells in an epigenetic drug model. Sci. Rep. 7, 44570. 10.1038/srep44570 28300163 PMC5353687

[B55] Ingber D. E. (2022). Human organs-on-chips for disease modelling, drug development and personalized medicine. Nat. Rev. Genet. 23, 467–491. 10.1038/s41576-022-00466-9 35338360 PMC8951665

[B56] Janani G. Priya S. Dey S. Mandal B. B. (2022). Mimicking native liver lobule microarchitecture *in vitro* with parenchymal and non-parenchymal cells using 3D bioprinting for drug toxicity and drug screening applications. ACS Appl. Mater Interfaces 14, 10167–10186. 10.1021/acsami.2c00312 35171571

[B57] Jang K. J. Otieno M. A. Ronxhi J. Lim H. K. Ewart L. Kodella K. R. (2019). Reproducing human and cross-species drug toxicities using a Liver-Chip. Sci. Transl. Med. 11, eaax5516. 10.1126/scitranslmed.aax5516 31694927

[B58] Jang M. Kim H. N. (2023). From single-to multi-organ-on-a-chip system for studying metabolic diseases. BioChip J. 17, 133–146. 10.1007/s13206-023-00098-z

[B59] Jessop Z. M. Al-Sabah A. Gardiner M. D. Combellack E. Hawkins K. Whitaker I. S. (2017). 3D bioprinting for reconstructive surgery: principles, applications and challenges. J. Plast. Reconstr. Aes Surg. 70, 1155–1170. 10.1016/j.bjps.2017.06.001 28734756

[B60] Jin X. Luong T. L. Reese N. Gaona H. Collazo-Velez V. Vuong C. (2014). Comparison of MDCK-MDR1 and Caco-2 cell based permeability assays for anti-malarial drug screening and drug investigations. J. Pharmacol. Toxicol. Methods 70, 188–194. 10.1016/j.vascn.2014.08.002 25150934

[B61] Jones A. W. (2011). Early drug discovery and the rise of pharmaceutical chemistry. Drug Test. Anal. 3, 337–344. 10.1002/dta.301 21698778

[B63] Kapałczyńska M. Kolenda T. Przybyła W. Zajączkowska M. Teresiak A. Filas V. (2016). 2D and 3D cell cultures – a comparison of different types of cancer cell cultures. Arch. Med. Sci. 14, 910–919. 10.5114/aoms.2016.63743 30002710 PMC6040128

[B153] Kasturi M. Vasanthan K. S. (2024). Harvesting decellularized liver extracellular matrix from rodents for 3D scaffold fabrication. Artif. Cell. Nanomed. Biotech. 52 (1), 175–185. 10.1080/21691401.2024.2319893 38423125

[B65] Khoury R. E. Ramirez S. P. Loyola C. D. Joddar B. (2023). Demonstration of doxorubicin’s cardiotoxicity and screening using a 3D bioprinted spheroidal droplet-based system. RSC Adv. 13, 8338–8351. 10.1039/D3RA00421J 36922946 PMC10010162

[B66] Kim J. Kim Y. Choi J. Jung H. Lee K. Kang J. (2018). Recapitulation of methotrexate hepatotoxicity with induced pluripotent stem cell-derived hepatocytes from patients with rheumatoid arthritis. Stem Cell Res. and Ther. 9, 357–415. 10.1186/s13287-018-1100-1 30594247 PMC6310944

[B67] Kim J. Kong J. S. Han W. Kim B. S. Cho D.-W. (2020). 3D cell printing of tissue/organ-mimicking constructs for therapeutic and drug testing applications. Int. J. Mol. Sci. 21, 7757. 10.3390/ijms21207757 33092184 PMC7589604

[B68] Kirillova A. Bushev S. Abubakirov A. Sukikh G. (2020). Bioethical and legal issues in 3D bioprinting. Int. J. Bioprinting 6, 272. 10.18063/ijb.v6i3.272 PMC7557521 33088986

[B69] Kloprogge F. Hammond R. Kipper K. Gillespie S. H. Della Pasqua O. (2019). Mimicking *in-vivo* exposures to drug combinations *in-vitro*: anti-tuberculosis drugs in lung lesions and the hollow fiber model of infection. Sci. Rep. 9, 13228. 10.1038/s41598-019-49556-5 31519935 PMC6744479

[B70] Ku J. Seonwoo H. Park S. Jang K.-J. Lee J. Lee M. (2020). Cell-laden thermosensitive chitosan hydrogel bioinks for 3D bioprinting applications. Appl. Sci. 10, 2455. 10.3390/app10072455

[B71] Lage O. M. Ramos M. C. Calisto R. Almeida E. Vasconcelos V. Vicente F. (2018). Current screening methodologies in drug discovery for selected human diseases. Mar. Drugs 16 (8), 279. 10.3390/md16080279 30110923 PMC6117650

[B72] Lai B. F. L. Lu R. X. Z. Hu Y. Davenport Huyer L. Dou W. Wang E. Y. (2020). Recapitulating pancreatic tumor microenvironment through synergistic use of patient organoids and organ‐on‐a‐chip vasculature. Adv. Funct. Mater 30, 2000545. 10.1002/adfm.202000545 33692660 PMC7939064

[B73] Laschke M. W. Menger M. D. (2017). Life is 3D: boosting spheroid function for tissue engineering. Trends Biotechnol. 35, 133–144. 10.1016/j.tibtech.2016.08.004 27634310

[B74] Lee C. Abelseth E. de la Vega L. Willerth S. M. (2019). Bioprinting a novel glioblastoma tumor model using a fibrin-based bioink for drug screening. Mater Today Chem. 12, 78–84. 10.1016/j.mtchem.2018.12.005

[B75] Lee J. A. Uhlik M. T. Moxham C. M. Tomandl D. Sall D. J. (2012). Modern phenotypic drug discovery is a viable, neoclassic pharma strategy. J. Med. Chem. 55, 4527–4538. 10.1021/jm201649s 22409666

[B76] Lee J. M. Sing S. L. Zhou M. Yeong W. Y. (2018). 3D bioprinting processes: a perspective on classification and terminology. Int. J. Bioprinting 4, 151. 10.18063/ijb.v4i2.151 PMC7582007 33102923

[B77] Levato R. Jungst T. Scheuring R. G. Blunk T. Groll J. Malda J. (2020). From shape to function: the next step in bioprinting. Adv. Mater. 32 (12), 1906423. 10.1002/adma.201906423 PMC7116209 32045053

[B78] Lind J. U. Busbee T. A. Valentine A. D. Pasqualini F. S. Yuan H. Yadid M. (2016). Instrumented cardiac microphysiological devices via multimaterial three-dimensional printing. Nat. Mater 16, 303–308. 10.1038/nmat4782 27775708 PMC5321777

[B79] Liu X. Michael S. Bharti K. Ferrer M. Song M. J. (2020). A biofabricated vascularized skin model of atopic dermatitis for preclinical studies. Biofabrication 12, 035002. 10.1088/1758-5090/ab76a1 32059197 PMC7457159

[B80] Loessner D. Stok K. S. Lutolf M. P. Hutmacher D. W. Clements J. A. Rizzi S. C. (2010). Bioengineered 3D platform to explore cell–ECM interactions and drug resistance of epithelial ovarian cancer cells. Biomaterials 31 (32), 8494–8506. 10.1016/j.biomaterials.2010.07.064 20709389

[B81] Loewa A. Feng J. J. Hedtrich S. (2023). Human disease models in drug development. Nat. Rev. Bioeng. 1, 545–559. 10.1038/s44222-023-00063-3 PMC10173243 37359774

[B82] Longati P. Jia X. Eimer J. Wagman A. Witt M. R. Rehnmark S. (2013). 3D pancreatic carcinoma spheroids induce a matrix-rich, chemoresistant phenotype offering a better model for drug testing. BMC Cancer 13, 95–13. 10.1186/1471-2407-13-95 23446043 PMC3617005

[B83] Longlong S. Haiqing B. Rodas M. Wuji C. Oh C. Y. Jiang A. (2021). A human-airway-on-a-chip for the rapid identification of candidate antiviral therapeutics and prophylactics. Nat. Biomed. Eng. 5, 815–829. 10.1038/s41551-021-00718-9 33941899 PMC8387338

[B154] Maia F. R. Reis R. L. Oliveira J. M. (2023). 3D-bioprinted in vitro disease models. In 3D printing in medicine. Woodhead Publishing, 179–198. 10.1016/B978-0-323-89831-7.00004-3

[B155] Mansouri M. Lam J. Sung K. E. (2024). Progress in developing microphysiological systems for biological product assessment. Lab on a Chip. 10.1039/D3LC00876B 38230512

[B84] Ma X. Yu C. Wang P. Xu W. Wan X. Lai C. S. E. (2018). Rapid 3D bioprinting of decellularized extracellular matrix with regionally varied mechanical properties and biomimetic microarchitecture. Biomaterials 185, 310–321. 10.1016/j.biomaterials.2018.09.026 30265900 PMC6186504

[B85] Mandrycky C. Wang Z. Kim K. Kim D.-H. (2016). 3D bioprinting for engineering complex tissues. Biotechnol. Adv. 34, 422–434. 10.1016/j.biotechadv.2015.12.011 26724184 PMC4879088

[B86] Maschmeyer I. Lorenz A. K. Schimek K. Hasenberg T. Ramme A. P. Hübner J. (2015). A four-organ-chip for interconnected long-term co-culture of human intestine, liver, skin and kidney equivalents. Lab. Chip 15, 2688–2699. 10.1039/C5LC00392J 25996126

[B87] Mastikhina O. Moon B. U. Williams K. Hatkar R. Gustafson D. Mourad O. (2020). Human cardiac fibrosis-on-a-chip model recapitulates disease hallmarks and can serve as a platform for drug testing. Biomaterials 233, 119741. 10.1016/j.biomaterials.2019.119741 31927251

[B88] Matsunaga N. Wada S. Nakanishi T. Ikenaga M. Ogawa M. Tamai I. (2014). Mathematical modeling of the *in vitro* hepatic disposition of mycophenolic acid and its glucuronide in sandwich-cultured human hepatocytes. Mol. Pharm. 11, 568–579. 10.1021/mp400513k 24320552

[B89] Mazrouei R. Velasco V. Esfandyarpour R. (2020). 3D-bioprinted all-inclusive bioanalytical platforms for cell studies. Sci. Rep. 10, 14669. 10.1038/s41598-020-71452-6 32887912 PMC7474064

[B90] Mazzocchi A. Soker S. Skardal A. (2019). 3D bioprinting for high-throughput screening: drug screening, disease modeling, and precision medicine applications. Appl. Phys. Rev. 6, 011302. 10.1063/1.5056188 33738018 PMC7968875

[B91] Mehta V. Vilikkathala Sudhakaran S. Nellore V. Madduri S. Rath S. N. (2024). 3D stem-like spheroids-on-a-chip for personalized combinatorial drug testing in oral cancer. J. Nanobiotechnol 22, 344. 10.1186/s12951-024-02625-y PMC11186147 38890730

[B92] Miller K. L. Xiang Y. Yu C. Pustelnik J. Wu J. Ma X. (2021). Rapid 3D bioprinting of a human iPSC-derived cardiac micro-tissue for high-throughput drug testing. Organs. Chip., 3:100007. 10.1016/j.ooc.2021.100007

[B93] Mittler F. Obeïd P. Rulina A. V. Haguet V. Gidrol X. Balakirev M. Y. (2017). High-Content monitoring of drug effects in a 3D spheroid model. Front. Oncol. 7, 293. 10.3389/fonc.2017.00293 29322028 PMC5732143

[B94] Mosaad E. O. Chambers K. F. Futrega K. Clements J. A. Doran M. R. (2018). The Microwell-mesh: a high-throughput 3D prostate cancer spheroid and drug-testing platform. Sci. Rep. 8, 253. 10.1038/s41598-017-18050-1 29321576 PMC5762676

[B95] Murphy C. Kolan K. Li W. Semon J. Day D. Leu M. (2017). 3D bioprinting of stem cells and polymer/bioactive glass composite scaffolds for bone tissue engineering. Int. J. Bioprinting 3, 53–63. 10.18063/ijb.2017.01.005 PMC7575634 33094180

[B96] Muwaffak Z. Goyanes A. Clark V. Basit A. W. Hilton S. T. Gaisford S. (2017). Patient-specific 3D scanned and 3D printed antimicrobial polycaprolactone wound dressings. Int. J. Pharm. 527 (1-2), 161–170. 10.1016/j.ijpharm.2017.04.077 28461267

[B156] Nam K. H. Smith A. S. Lone S. Kwon S. Kim D. H. (2015). Biomimetic 3D tissue models for advanced high-throughput drug screening. J. Labora. Automa. 20 (3), 201–215. 10.1177/2211068214557813 PMC4459652 25385716

[B97] Naghieh S. Sarker M. D. Abelseth E. Chen X. (2019). Indirect 3D bioprinting and characterization of alginate scaffolds for potential nerve tissue engineering applications. J. Mech. Behav. Biomed. Mater 93, 183–193. 10.1016/j.jmbbm.2019.02.014 30802775

[B98] Nel F. P. Rymajdo K. (2024). Securing the future of UK public-interest news: navigating change with foresight and innovation. Media Commun. 12. 10.17645/mac.7497

[B99] Newman D. J. Cragg G. M. (2012). Natural products as sources of new drugs over the 30 years from 1981 to 2010. J. Nat. Prod. 75, 311–335. 10.1021/np200906s 22316239 PMC3721181

[B100] Njoroge W. Hernández A. C. H. Musa F. I. Butler R. Harper A. G. S. Yang Y. (2021). The combination of tissue-engineered blood vessel constructs and parallel flow chamber provides a potential alternative to *in vivo* drug testing models. Pharmaceutics 13 (3), 340. 10.3390/pharmaceutics13030340 33807995 PMC7998107

[B101] Norona L. M. Nguyen D. G. Gerber D. A. Presnell S. C. LeCluyse E. L. (2016). Editor’s highlight: modeling compound-induced Fibrogenesis*In Vitro*Using three-dimensional bioprinted human liver tissues. Toxicol. Sci. 154, 354–367. 10.1093/toxsci/kfw169 27605418 PMC5139067

[B102] Pagnotta G. Kalia S. Di Lisa L. Cicero A. F. G. Borghi C. Focarete M. L. (2022). Progress towards 3D bioprinting of tissue models for advanced drug screening: *in vitro* evaluation of drug toxicity and drug metabolism. Bioprinting 27, e00218. 10.1016/j.bprint.2022.e00218

[B103] Palaninathan V. Kumar V. Maekawa T. Liepmann D. Paulmurugan R. Eswara J. R. (2018). Multi-organ on a chip for personalized precision medicine. MRS Commun. 8, 652–667. 10.1557/mrc.2018.148

[B104] Pampaloni F. Reynaud E. G. Stelzer E. H. (2007). The third dimension bridges the gap between cell culture and live tissue. Nat. Rev. Mol. Cell Biol. 8, 839–845. 10.1038/nrm2236 17684528

[B105] Pati F. Jang J. Ha D.-H. Won Kim S. Rhie J.-W. Shim J.-H. (2014). Printing three-dimensional tissue analogues with decellularized extracellular matrix bioink. Nat. Commun. 5, 3935. 10.1038/ncomms4935 24887553 PMC4059935

[B106] Peng W. Datta P. Ayan B. Ozbolat V. Sosnoski D. Ozbolat I. T. (2017). 3D bioprinting for drug discovery and development in pharmaceutics. Acta Biomater. 57, 26–46. 10.1016/j.actbio.2017.05.025 28501712

[B107] Phang S. J. Basak S. Teh H. X. Packirisamy G. Fauzi M. B. Kuppusamy U. R. (2022). Advancements in extracellular matrix-based biomaterials and biofabrication of 3D organotypic skin models. ACS Biomater. Sci. Eng. 8, 3220–3241. 10.1021/acsbiomaterials.2c00342 35861577

[B108] Pickl M. Ries C. H. (2009). Comparison of 3D and 2D tumor models reveals enhanced HER2 activation in 3D associated with an increased response to trastuzumab. Oncogene 28, 461–468. 10.1038/onc.2008.394 18978815

[B109] Picollet-D’hahan N. Zuchowska A. Lemeunier I. Le Gac S. (2021). Multiorgan-on-a-Chip: a systemic approach to model and decipher inter-organ communication. Trends Biotechnol. 39, 788–810. 10.1016/j.tibtech.2020.11.014 33541718

[B110] Pieterman E. D. Den V. Der V. Svensson E. M. Bax H. I. de M. (2021). Higher dosing of rifamycins does not increase activity against *Mycobacterium tuberculosis* in the hollow-fiber infection model. Antimicrob. Agents Chemother. 65. 10.1128/aac.02255-20 PMC8097456 33558283

[B111] Pothysvaran S. Balachander S. Ashwini S. (2024). “Legal and bioethical view of educational sectors and industrial areas of 3D bioprinting,” in Computational intelligence in bioprinting, eds Gangadevi E. Shri M. L. Dhanaraj R. K. Baluswamy B. Wiley 127–155.

[B112] Ramesh S. Deep A. Tamayol A. Kamaraj A. Mahajan C. Madihally S. (2024). Advancing 3D bioprinting through machine learning and artificial intelligence. Bioprinting 38, e00331. 10.1016/j.bprint.2024.e00331

[B113] Ravi M. Paramesh V. Kaviya S. R. Anuradha E. Solomon F. D. (2015). 3D cell culture systems: advantages and applications. J. Cell Physiol. 230, 16–26. 10.1002/jcp.24683 24912145

[B114] Rizwana N. Maslekar N. Chatterjee K. Yao Y. Agarwal V. Nune M. (2023). Dual crosslinked antioxidant mixture of poly(vinyl alcohol) and cerium oxide nanoparticles as a bioink for 3D bioprinting. ACS Appl. Nano Mater 7, 18177–18188. 10.1021/acsanm.3c02962 39206348 PMC11348314

[B115] Rous P. A. (1910). A transmissible avian neoplasm (sarcoma of the common fowl). J. Exp. Med. 12, 696–705. 10.1084/jem.12.5.696 19867354 PMC2124810

[B116] Satpathy A. Datta P. Wu Y. Ayan B. Bayram E. Ozbolat I. T. (2018). Developments with 3D bioprinting for novel drug discovery. Expert Opin. Drug Discov. 13, 1115–1129. 10.1080/17460441.2018.1542427 30384781 PMC6494715

[B117] Schaeffner I. Petters J. Aurich H. Frohberg P. Christ B. (2005). A microtiterplate-based screening assay to assess diverse effects on cytochrome P450 enzyme activities in primary rat hepatocytes by various compounds. Assay. Drug Dev. Technol. 3, 27–38. 10.1089/adt.2005.3.27 15798393

[B118] Schlabach M. R. Luo J. Solimini N. L. Hu G. Xu Q. Li M. Z. (2008). Cancer proliferation gene discovery through functional genomics. Science 319, 620–624. 10.1126/science.1149200 18239126 PMC2981870

[B119] Scognamiglio G. De Chiara A. Parafioriti A. Armiraglio E. Fazioli F. Gallo M. (2019). Patient-derived organoids as a potential model to predict response to PD-1/PD-L1 checkpoint inhibitors. Br. J. cancer 121 (11), 979–982. 10.1038/s41416-019-0616-1 31666667 PMC6889147

[B120] Shin S. R. Kilic T. Zhang Y. S. Avci H. Hu N. Kim D. (2017). Label-free and regenerative electrochemical microfluidic biosensors for continual monitoring of cell secretomes. Adv. Sci. 4, 1600522. 10.1002/advs.201600522 PMC5441508 28546915

[B121] Singh N. Vayer P. Tanwar S. Poyet J.-L. Tsaioun K. Villoutreix B. O. (2023). Drug discovery and development: introduction to the general public and patient groups. Front. Drug Discov. 3, 1201419. 10.3389/fddsv.2023.1201419

[B122] Singh Y. Joseph C. M. Bandyopadhyay A. Mandal B. B. (2022). 3D bioprinted silk‐based *in vitro* osteochondral model for osteoarthritis therapeutics. Adv. Healthc. Mater 11, 2200209. 10.1002/adhm.202200209 35670084

[B123] Song D. Xu Y. Liu S. Wen L. Wang X. (2021). Progress of 3D bioprinting in organ manufacturing. Polymers 13, 3178. 10.3390/polym13183178 34578079 PMC8468820

[B124] Srivastava S. Pasipanodya G. J. Ramachandran G. Deshpande D. Shuford S. Crosswell H. E. (2016). A long-term Co-perfused disseminated tuberculosis-3D liver hollow fiber model for both drug efficacy and hepatotoxicity in babies. EBioMedicine 6, 126–138. 10.1016/j.ebiom.2016.02.040 27211555 PMC4856747

[B125] Sun L. Yang H. Wang Y. Zhang X. Jin B. Xie F. (2020). Application of a 3D bioprinted hepatocellular carcinoma cell model in antitumor drug research. Front. Oncol. 10, 878. 10.3389/fonc.2020.00878 32582546 PMC7283506

[B126] Tan B. Gan S. Wang X. Liu W. Li X. (2021). Applications of 3D bioprinting in tissue engineering: advantages, deficiencies, improvements, and future perspectives. J. Mater Chem. B 9, 5385–5413. 10.1039/d1tb00172h 34124724

[B127] Tang M. Tiwari S. K. Agrawal K. Tan M. Dang J. Tam T. (2021). Rapid 3D bioprinting of glioblastoma model mimicking native biophysical heterogeneity. Small 17, 2006050. 10.1002/smll.202006050 PMC8049977 33502104

[B128] Tebon P. J. Wang B. Markowitz A. L. Davarifar A. Tsai B. L. Krawczuk P. (2023). Drug screening at single-organoid resolution via bioprinting and interferometry. Nat. Commun. 14, 3168. 10.1038/s41467-023-38832-8 37280220 PMC10244450

[B129] Thompson A. (2023). 3D bioprinting platform offers realistic cardiac tissue model for drug testing. Scilight 2023 (48). 10.1063/10.0023844

[B130] Tian T. Ho Y. Chen C. Sun H. Hui J. Yang P. (2022). A 3D bio-printed spheroids-based perfusion *in vitro* liver on chip for drug toxicity assays. Chin. Chem. Lett. 33, 3167–3171. 10.1016/j.cclet.2021.11.029

[B131] Vlachogiannis G. Hedayat S. Vatsiou A. Jamin Y. Fernández-Mateos J. Khan K. (2018). Patient-derived organoids model treatment response of metastatic gastrointestinal cancers. Sci. (New York, N.Y.) 359 (6378), 920–926. 10.1126/science.aao2774 PMC6112415 29472484

[B132] Votanopoulos K. I. Forsythe S. Sivakumar H. Mazzocchi A. Aleman J. Miller L. (2019). Model of patient-specific immune-enhanced organoids for immunotherapy screening: feasibility study. Ann. Surg. Oncol. 27, 1956–1967. 10.1245/s10434-019-08143-8 31858299 PMC7474462

[B133] Wang H. F. Ran R. Liu Y. Hui Y. Zeng B. Chen D. (2018a). Tumor-vasculature-on-a-chip for investigating nanoparticle extravasation and tumor accumulation. ACS nano 12 (11), 11600–11609. 10.1021/acsnano.8b06846 30380832

[B134] Wang L. Dou W. Malhi M. Zhu M. Liu H. Plakhotnik J. (2018b). Microdevice platform for continuous measurement of contractility, beating rate, and beating rhythm of human-induced pluripotent stem cell-cardiomyocytes inside a controlled incubator environment. ACS Appl. Mater Interfaces 10, 21173–21183. 10.1021/acsami.8b05407 29874032

[B135] Wen Z. Liao Q. Hu Y. You L. Zhou L. Zhao Y. (2013). A spheroid-based 3-D culture model for pancreatic cancer drug testing, using the acid phosphatase assay. Braz J. Med. Biol. Res. 46, 634–642. 10.1590/1414-431X20132647 23903680 PMC3859338

[B136] Wheeler G. Field R. Tomlinson M. (2012). “Phenotypic screens with model organisms,” in Chemical genomics. Editor Fu H. (New York: Cambridge University Press), 121–136.

[B137] Wu Y. Heikal L. Ferns G. Ghezzi P. Nokhodchi A. Maniruzzaman M. (2019). 3D bioprinting of novel biocompatible scaffolds for endothelial cell repair. Polymers 11, 1924. 10.3390/polym11121924 31766610 PMC6960937

[B138] Yadid M. Lind J. U. Ardoña H. A. M. Sheehy S. P. Dickinson L. E. Eweje F. (2020). Endothelial extracellular vesicles contain protective proteins and rescue ischemia-reperfusion injury in a human heart-on-chip. Sci. Transl. Med. 12, eaax8005. 10.1126/scitranslmed.aax8005 33055246 PMC8969368

[B139] Yang K. Guo C. Woodhead J. L. St Claire R. L. Watkins P. B. Siler S. Q. (2016). Sandwich-cultured hepatocytes as a tool to study drug disposition and drug-induced liver injury. J. Pharm. Sci. 105, 443–459. 10.1016/j.xphs.2015.11.008 26869411 PMC4894499

[B140] Yang Y. Xu R. Wang C. Guo Y. Sun W. Ouyang L. (2022). Recombinant human collagen-based bioinks for the 3D bioprinting of full-thickness human skin equivalent. Int. J. Bioprinting 8, 611. 10.18063/ijb.v8i4.611 PMC9668586 36404779

[B141] Yi H.-G. Jeong Y. H. Kim Y. Choi Y.-J. Moon H. E. Park S. H. (2019). A bioprinted human-glioblastoma-on-a-chip for the identification of patient-specific responses to chemoradiotherapy. Nat. Biomed. Eng. 3, 509–519. 10.1038/s41551-019-0363-x 31148598

[B142] Yin J. Yan M. Wang Y. Fu J. Suo H. (2018). 3D bioprinting of low-concentration cell-laden gelatin methacrylate (GelMA) bioinks with a two-step cross-linking strategy. ACS Appl. Mater Interfaces 10, 6849–6857. 10.1021/acsami.7b16059 29405059

[B143] Yoshii Y. Furukawa T. Aoyama H. Adachi N. Zhang M.-R. Hidekatsu W. (2016). Regorafenib as a potential adjuvant chemotherapy agent in disseminated small colon cancer: drug selection outcome of a novel screening system using nanoimprinting 3-dimensional culture with HCT116-RFP cells. Int. J. Oncol. 48, 1477–1484. 10.3892/ijo.2016.3361 26820693

[B144] Zamani Y. Mohammadi J. Amoabediny G. Helder M. N. Zandieh-Doulabi B. Klein-Nulend J. (2020). Bioprinting of alginate-encapsulated pre-osteoblasts in PLGA/β-TCP scaffolds enhances cell retention but impairs osteogenic differentiation compared to cell seeding after 3D-printing. Regen. Eng. Transl. Med. 7, 485–493. 10.1007/s40883-020-00163-1

[B145] Zhang B. Luo Y. Ma L. Gao L. Li Y. Xue Q. (2018). 3D bioprinting: an emerging technology full of opportunities and challenges. Bio-Des Manuf. 1, 2–13. 10.1007/s42242-018-0004-3

[B146] Zhang X. Abutaleb N. O. Salmon E. Truskey G. A. (2022). *In situ* fabrication and perfusion of tissue-engineered blood vessel microphysiological system. Methods Mol. Biol. Clift. N.J. 2375, 77–90. 10.1007/978-1-0716-1708-3_7 PMC9375439 34591300

[B147] Zheng F. Fu F. Cheng Y. Wang C. Zhao Y. Gu Z. (2016). Organ-on-a-Chip systems: microengineering to biomimic living systems. Small 12, 2253–2282. 10.1002/smll.201503208 26901595

[B148] Zhou S. F. Zhong W. Z. (2017). Drug design and discovery: principles and applications. Mol. Basel, Switz. 22 (2), 279. 10.3390/molecules22020279 PMC6155886 28208821

[B149] Zhou X. Zhu W. Nowicki M. Miao S. Cui H. Holmes B. (2016). 3D bioprinting a cell-laden bone matrix for breast cancer metastasis study. ACS Appl. Mater Interfaces 8, 30017–30026. 10.1021/acsami.6b10673 27766838

[B150] Zhu Y. Mandal K. Hernandez A. L. Kawakita S. Huang W. Bandaru P. (2021). State of the art in integrated biosensors for organ-on-a-chip applications. Curr. Opin. Biomed. Eng. 19, 100309. 10.1016/j.cobme.2021.100309 37206309 PMC10193909

[B151] Zollinger A. J. Smith M. L. (2017). Fibronectin, the extracellular glue. Matrix Biol. 60–61, 27–37. 10.1016/j.matbio.2016.07.011 27496349

